# Construction of Core Collections Suitable for Association Mapping to Optimize Use of Mediterranean Olive (*Olea europaea* L.) Genetic Resources

**DOI:** 10.1371/journal.pone.0061265

**Published:** 2013-05-07

**Authors:** Ahmed El Bakkali, Hicham Haouane, Abdelmajid Moukhli, Evelyne Costes, Patrick Van Damme, Bouchaib Khadari

**Affiliations:** 1 INRA, UMR Amélioration Génétique et Adaptation des Plantes (AGAP), Montpellier, France; 2 Montpellier SupAgro, UMR AGAP, Montpellier, France; 3 INRA Meknès, UR Amélioration des Plantes et Conservation des Ressources Phytogénétiques, Meknès, Morocco; 4 Department of Plant Production, Ghent University, Ghent, Belgium; 5 INRA Marrakech, UR Amélioration des Plantes, Marrakech, Morocco; 6 Institute of Tropics and Subtropics, Czech University of Life Sciences Prague, Prague, Czech Republic; 7 Conservatoire Botanique National Méditerranéen, UMR AGAP, Montpellier, France; United States Department of Agriculture, United States of America

## Abstract

Phenotypic characterisation of germplasm collections is a decisive step towards association mapping analyses, but it is particularly expensive and tedious for woody perennial plant species. Characterisation could be more efficient if focused on a reasonably sized subset of accessions, or so-called core collection (CC), reflecting the geographic origin and variability of the germplasm. The questions that arise concern the sample size to use and genetic parameters that should be optimized in a core collection to make it suitable for association mapping. Here we investigated these questions in olive (*Olea europaea* L.), a perennial fruit species. By testing different sampling methods and sizes in a worldwide olive germplasm bank (OWGB Marrakech, Morocco) containing 502 unique genotypes characterized by nuclear and plastid loci, a two-step sampling method was proposed. The Shannon-Weaver diversity index was found to be the best criterion to be maximized in the first step using the *Core Hunter* program. A primary core collection of 50 entries (CC_50_) was defined that captured more than 80% of the diversity. This latter was subsequently used as a kernel with the *Mstrat* program to capture the remaining diversity. 200 core collections of 94 entries (CC_94_) were thus built for flexibility in the choice of varieties to be studied. Most entries of both core collections (CC_50_ and CC_94_) were revealed to be unrelated due to the low kinship coefficient, whereas a genetic structure spanning the eastern and western/central Mediterranean regions was noted. Linkage disequilibrium was observed in CC_94_ which was mainly explained by a genetic structure effect as noted for OWGB Marrakech. Since they reflect the geographic origin and diversity of olive germplasm and are of reasonable size, both core collections will be of major interest to develop long-term association studies and thus enhance genomic selection in olive species.

## Introduction

Recent advances in genomic tools, including genome sequencing [1] and high-density single nucleotide polymorphism (SNP) genotyping [2], and statistical methods have enabled the development of new approaches for mapping of complex traits. The identification of causal genes underlying specific traits is a major goal in plant breeding, subsequently offering opportunities to develop genomic selection tools [3]–[4]. Association mapping (also known as linkage disequilibrium (LD)-based association mapping) [5] has been proposed to associate single DNA sequence changes with traits of interest using collections of unrelated individuals, as an alternative or complement to quantitative trait locus (QTL)-mapping (also known as family-based linkage mapping) [6]. Association mapping has been largely documented and successfully used to identify the genetic basis of many complex diseases in humans [7], and is now emerging in plants [8]–[9]. It has the advantage of being rapid and cost effective as many alleles may be assessed simultaneously, resulting in higher resolution mapping by the use of most recombination events that occur over time, while avoiding the need to expensively and tediously develop crossing populations, particularly for perennial and forest tree species [10]. The number of markers needed to map specific associations depends on the extent and distribution of LD within the species and among linkage groups [5]. Many studies have thus proposed an estimate of LD in different plant species as a preliminary step for association analysis [11]–[14]. Association mapping results obtained in a number of annual species, e.g. *Arabidopsis thaliana* [15]–[16], *Oryza sativa* [17]–[18], *Triticum aestivum* [19] and *Zea mays* [20]–[21], indicate that the approach is promising to identify markers correlated with desirable traits such as flowering time [15]–[16], [20], seed morphology [19], [22] and disease resistance [15], [23]–[24]. However, for woody and perennial species, studies have been performed on a limited number of species, such as *Pinus taeda* L. [25], *Eucalyptus* spp. [26] and *Prunus persica* [27].

Beyond the importance of *ex situ* conservation of genetic resources to avoid genetic erosion and provide plant breeders with easy access to study ranges of variation in phenotypic traits, germplasm collections could serve as a reservoir of outstanding genes to enhance agronomic traits so as to meet the needs of diverse agricultural systems. However, field evaluation and use of large germplasm collections for association mapping purposes are mostly constrained by problems of accession redundancy, economic cost and time, especially for clonally propagated perennial species where clones have to be maintained and evaluated for several years at different sites. Genetic resource assessments could thus be more rational if focused on a subset of accessions, or so-called core collection (CC; also known as core subset), which includes in the sample as much variability present in the whole collection as possible with minimal size [28]. Determining the best sample size to use and genetic criteria to be optimized for association mapping in one core collection is an open issue requiring further investigation, especially for perennial species. Over the last decade, several core subsets have been proposed for both annual species, e.g. *Arabidopsis thaliania* [29], *Oryza sativa* [30], *Triticum aestivum* [31] and *Zea mays* [32], and perennial species, e.g. *Annona cherimola* [33], *Malus domestica* [34], *Prunus armeniaca* [35] and *Vitis vinifera* [36], using different eco-geographical, agro-morphological, biochemical or molecular data. Despite the many approaches used to design core collections that optimize the genetic distance between accessions and/or the allelic diversity [37]–[44], most of core collections have been constructed based on the so-called maximizing method (M-method) [37] through the *Mstrat* program [40] by optimizing the number of alleles/trait classes for germplasm conservation purposes, whereas core sizes depend on the number of accessions and the diversity available in the base collections. Sample sizes of 5–20% of the whole collection, encompassing at least 70% of observed alleles, were considered optimal in many studies [45]–[46].

Olive, which is one of the most important fruit crops in the Mediterranean area [47], is cultivated in more than 24 countries, whereas more than 1200 olive varieties have been reported [48]–[49] and conserved in many germplasm collections around the world [50], including two worldwide olive germplasm banks (OWGB) in Cordoba (Spain) [51] and Marrakech (Morocco) [52]. The available diversity has been evaluated using morphological descriptors and diverse molecular markers (AFLP, SSR, SNPs, DArt) [53]–[58]. However, only a few cross-breeding programs make use of olive germplasm for QTL mapping [59] as many constraints currently hinder the development of bi-parental populations, i.e. a long juvenile period [60], low fruit set [61], low seed germination [62] and lack of knowledge about trait heritability [63]–[65]. LD-based association mapping is thus considered to be a suitable approach to determine the genetic basis of traits in olive varieties according to the available diversity. Moreover, the development of a core collection is thus essential to effectively optimize the use of such diversity. Two core collections encompassing total allelic diversity of OWGB Cordoba have currently been reported [51], [66]. However, only a single core collection was proposed in each study, which hinders effective and flexible use of the broad range of olive diversity, and western Mediterranean accessions, particularly those originating from Spain (more than 40% of entries in the CC), are over-represented in both core collections. In addition, despite using two different sampling algorithms via *Mstrat* [40] and *Core Hunter* [43] programs, these core collections were developed based only on capturing total alleles (or allelic coverage; *Cv*) as main criterion, which is questionable for sampling as it excludes selection of highly genetically distant entries, whereas both core collections were not investigated regarding the genetic structure and relatedness between selected entries for association mapping.

Here a two-step method using nuclear microsatellite loci, *cpDNA* haplotypes and agro-morphological traits is proposed, combining the assets of *Mstrat* and *Core Hunter* programs, with the aim of building flexible olive core collections from OWGB Marrakech suitable for association studies. We specifically aimed to (1) compare various sampling methods and sizes to select the best ones based on diverse criteria, and (2) propose many core collections with optimal sizes for field evaluation and which reflect the geographic and diversity of olive. The convenience of the developed core collections for association mapping is examined with regard to genetic structure, relatedness and linkage disequilibrium.

## Materials and Methods

### Dataset

A total of 561 accessions from 14 countries, maintained in the *ex situ* OWGB Marrakech collection, were used in this study (Table S1). A set of 17 SSR loci was used for accession genotyping (Text S1). Plastid DNA (or *cpDNA*) was characterized using 37 polymorphic loci and two cleaved amplified polymorphism sites (CAPS-*XapI* and CAPS-*EcorRI)*, as described by Besnard et al. [67] (Text S1).

The phenotypic data was from olive databases and national catalogues based on passport data and variety name as identification key [68]–[72]. Data on 72 agro-morphological traits classified into 213 trait classes according to standards described by the International Olive Oil Council (IOOC) was compiled for 425 varieties (Table S2).

### Construction of Core Subsets

**Figure 1:**
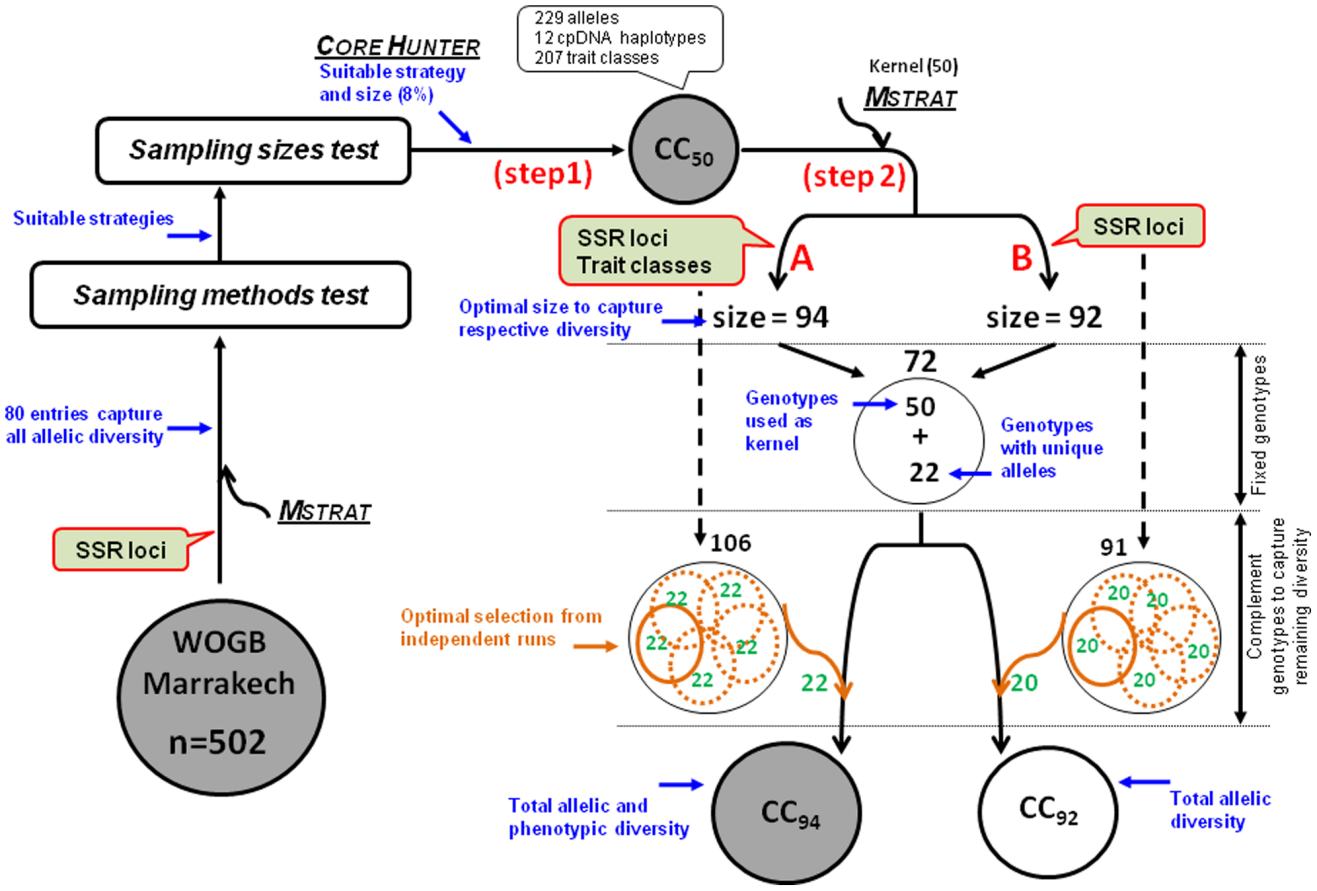
Current study flow chart to construct core collections from OWGB Marrakech. There were two main steps. As a benchmark, a sample size was determined using the *Mstrat* program to compare different sampling methods and sizes; 80 entries were necessary to capture all alleles. A primary core collection (CC_50_) was constructed using the *Core Hunter* program at 8% sample size (step 1). Then CC_50_ was used as a kernel to select the minimum size required to capture the total diversity using the *Mstrat* program (step 2). At this step, two procedures were performed, i.e. sampling with nuclear markers and trait classes (A; 94 entries were necessary) or using only nuclear markers (B; 92). For both procedures, a set of 72 genotypes was used in all independent runs while a combination of 22 complement genotypes could be selected from a panel of 106 genotypes to capture all of the allelic and phenotypic diversity (CC_94_) or 20 genotypes from a panel of 91 genotypes to capture the total allelic diversity (CC_92_).

To compare the performance of current state-of-the-art methods to construct core subsets, as a benchmark, we estimated the minimum size necessary to capture all the observed alleles using the *Mstrat* program (Figure 1). The size assessment indicated that 80 entries were necessary to capture the total allelic diversity (16% of OWGB Marrakech). Then, at this sample size, four different sampling methods were first tested:

1. The maximizing method (M method) implemented in the *Mstrat* program. By using an iterative maximization procedure, *Mstrat* examines all possible core subsets and singles out those that maximize the number of alleles (and/or trait classes) in dataset for one sample size. The program allows to specify a compulsory set of accessions, called a “kernel”, that will always be included in the core subset. In this case, maximization was focused on complementing alleles not included in the kernel. The Shannon-Weaver diversity index [73] was used as a second criterion to classify core subsets capturing the same number of alleles.
2. The advanced stochastic local search method (ASLS method) implemented in the *Core Hunter* program. The program is able to select core subsets using diverse allocation strategies by optimizing one genetic parameter or many parameters simultaneously, whereby the best solution among all replicas is reported. For instance, optimizing only the genetic distance, i.e. “*D_CE_* strategy”, the proposed core subset typically consists of genetically distant accessions, whereas the “*Cv* strategy” emphasizes the selection of genotypes with the most diverse alleles. Three allocation strategies were used: (i) optimizing each of the following measures independently (average Cavalli-Sforza and Edwards genetic distance “*D_CE_* strategy” [74], allelic coverage or number of alleles “*Cv* strategy”, Shannon-Weaver diversity index “*Sh strategy”*, or Nei diversity index “*He strategy*” [75]); (ii) optimizing all measures simultaneously with equal weight assigned to each one “multi-strategy”; and (iii) optimizing both *D_CE_* and *Cv* simultaneously (“*D_CE_Cv* strategy”). A previous analysis revealed that when a weight of 60% was assigned to *D_CE_* and 40% to *Cv*, all observed alleles were captured in the sampled subset (Figure S1).
3. The maximum length sub-tree method (MLST method) implemented in the *DARwin* v.5.0.137 program [41]. Starting from a diversity tree, the procedure is performed step by step. At each step, the unit for each pair with the minimal length of the external edge in the tree is removed. The procedure searches for the most unstructured tree, i.e. a star-like tree, by successive pruning of redundant units. The genetic distance between genotypes was calculated using the sample matching coefficient [76] and the tree was drawn based on the Neighbor-joining method [77].
4. The random method (R-method) using the *PowerMarker* v.2.25 program [78]. Samples were selected arbitrarily without replacement of genotypes.

Moreover, four other sizes were tested by the optimal methods selected at 16% sample size, i.e. 4% (20 entries), 8% (40), 24% (120) and 32% (160). To simplify the notation, we assigned a code to each sampled subset, as shown in Table 1 and in Table S3. For instance, CC1-80 is the subset sampled at 16% sample size (80 entries) using the “*Cv* strategy” with the ASLS method. Twenty replicates and 100 iterations were generated independently for each sample size and method without prior knowledge of the origin of the respective varieties. Once the optimal sampling method and size were selected, two procedures were performed in the second sampling step: (i) sampling with both nuclear markers and agro-morphological traits and (ii) using only nuclear markers (Figure 1). These procedures were compared in order to test the effect of using phenotypic traits when sampling entries. In addition, 14 reference varieties were considered significant when constructing the core subsets. These varieties were considered to be the most prominent and most cultivated in the olive-growing Mediterranean countries as well as being commonly involved in olive breeding programs: “Leccino”, “Frantoio” and “Carolea” (from Italy), “Picual” and “Hojiblanca” (Spain), “Galega vulgar” (Portugal), “Zaity” (Syria), “Picholine Marocaine” (Morocco), “Chetoui” (Tunisia), “Koroneiki” and “Amphisis” (Greece), “Aggizi Shami” (Egypt), “Chemlal de Kabylie” (Algeria), and “Picholine de Languedoc” (France).

**Table 1:** Genetic parameters of core subsets selected by different sampling methods at 16% sample size: advanced stochastic local search (ASLS), maximizing (M), maximum length sub-tree (MLST) and random (R).

Subset Code	Method/allocation strategy	*Cv (%)*	*D_CE_* (±SD)	*He*	*Sh*	*# Trait classes (%)*	# haplotypes
	OWGB Marrakech	279	0.746 (±0.092)	0.728	4.524	213	12
CC1-80	ASLS/Cv^1^	279 (100)	0.793 (±0.076)	0.77	4.731	206 (96.7)	12 (100)
**CC2-80**	**ASLS/D_CE_** 1	**234 (84)**	**0.833 (±0.07)**	**0.808** *	**4.829**	**202 (94.8)**	**11 (91.6)**
**CC3-80**	**ASLS/He** 1	**232 (83)**	**0.828 (±0.067)**	**0.814** *	**4.839**	**201 (94.3)**	**11 (91.6)**
**CC4-80**	**ASLS/Sh** 1	**250 (89.6)**	**0.825 (±0.068)**	**0.807** *	**4.861**	**204 (95.7)**	**11 (91.6)**
**CC5-80**	**ASLS/multi** 2	**265 (95)**	**0.82 (±0.069)**	**0.799** *	**4.836**	**205 (96.2)**	**11 (91.6)**
CC6-80	ASLS/D_CE_Cv^3^	279 (100)	0.806 (±0.071)	0.779	4.773	205 (96.2)	11 (91.6)
CC7-80	M	279 (100)	0.804 (±0.07)	0.786	4.773	204 (95.77)	12 (100)
CC8-80	MLST	236 (84.6)	0.817 (±0.061)	0.797*	4.778	205 (96.2)	10 (83.3)
CC9-80	R	202 (72.4)*	0.749 (±0.097)	0.731	4.507	199 (93.4)	10 (83.3)

Four sampling strategies using the ASLS method were found to be the most suitable for comparing different sampling sizes (in bold).

*Cv:* allelic coverage or number of alleles, *D_CE_:* average genetic distance of Cavalli-Sforza and Edwards, *SD*: standard deviation, *He*: Nei diversity index, *Sh*: Shannon-Weaver diversity index.

1 Each parameter was optimized independently by performing 20 runs with 100% weight given to the respective parameters (“*Cv* strategy”, “*D_CE_*”, “*Sh*”, and “*He*”).

2 Twenty independent runs were performed with equal weight given to each of the four parameters simultaneously (“multi strategy”).

3 Subset sampled when a weight of 60% was assigned to *D_CE_* and 40% to *Cv* (“*D_CE_Cv* strategy”).

* Statistically significant difference (*p<0.05*) using the Mann-Whitney test between core subsets and OWGB Marrakech.

### Comparison of Sampling Methods and Sample Sizes

To test the ability of each sampling method and size in capturing the diversity and representativeness in the sampled subset as compared to OWGB Marrakech, different criteria were considered: (i) the recovery of maximum alleles, trait classes and *cpDNA* haplotypes observed in the whole collection; (ii) a high and significant Shannon-Weaver diversity index estimated by the *t-*test (*p≤*0.05); (iii) no significant differences in the Nei diversity index and in allelic richness computed by the Mann-Whitney test (*p≤*0.05) with the *Past* program [79]; and (iv) the presence of the 14 reference varieties defined above.

### Assessment of Core Collections for Association Mapping Purposes

As the sub-structure within subsets and the relatedness between genotypes (known also as the kinship coefficient) are the major components to take into consideration in association mapping analyses [80]–[82], an assessment of both factors in proposed core collections was performed. Two approaches were used to assess the genetic structure; (i) principal coordinate analysis (PCoA) implemented in the *DARwin* v.5.0.137 program using a simple matching coefficient to describe the spatial distribution of genotypes; and (ii) model-based Bayesian clustering implemented in *Structure v.2.2* [83] according to the parameters described in Haouane et al. [52]. The reliability of the number of K clusters was checked using the ad-hoc ΔK measure [84] with the R program whereas the similarity index between 10 replicates for the same K clusters (*H*′) was calculated via *Clumpp* [85].

The relative kinship coefficient between genotypes was computed via *Spagedi* [86] through the coefficient of Loiselle et al. [87]. Negative values between two individuals, indicating that there was less relationship than that expected between two random individuals were replaced by 0, as proposed by Yu et al. [80]. The *Tassel* 2.0 program [88] was used to estimate the LD (*r^2^ coefficient*) among 17 nuclear loci after deletion of low frequency alleles (less than 0.05). A *p*-value for each LD score was computed through 1000 permutations to determine the significance. For the whole collection, only genotypes distinguished by more than three dissimilar alleles were considered when computing the kinship coefficient and LD in order to avoid considering variants of the same genotype.

## Results

### Characterization of Worldwide Olive Germplasm Bank of Marrakech

Using 17 nuclear SSR loci, all 561 accessions of OWGB Marrakech were classified into 502 distinct SSR profiles (Table S1) whereas 457 genotypes were distinguished by more than 3 dissimilar alleles. A total of 279 alleles were revealed with a mean of 16.4 alleles per locus (Text S2). The set of plastid markers revealed the presence of 12 haplotypes in OWGB Marrakech, with one highly frequent one (E1.1, 83.2%; Text S2).

### Comparison of Sampling Methods

**Figure 2:**
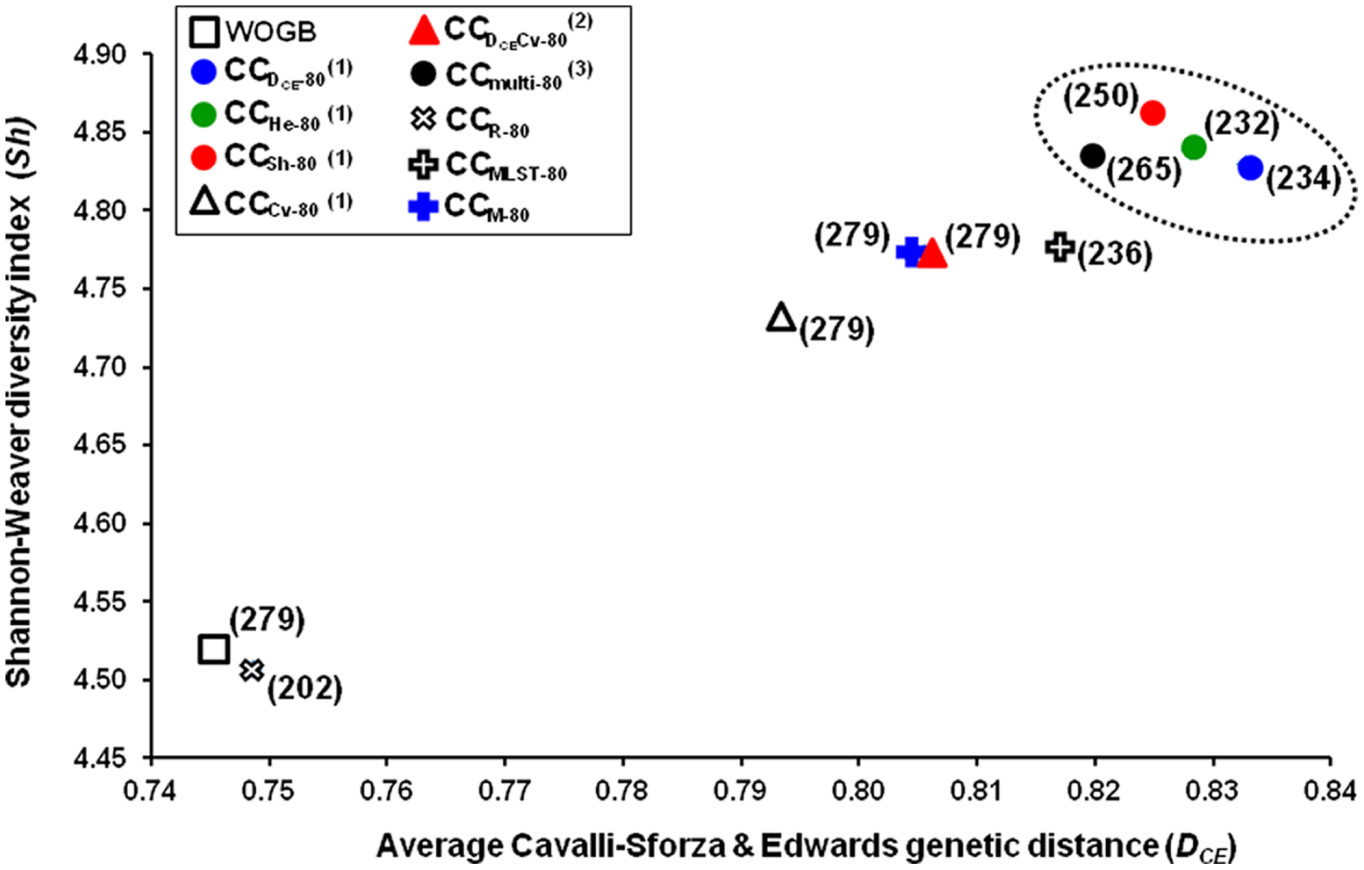
Comparison of sampling methods according to average genetic distance (*D_CE_*) and Shannon-Weaver diversity index (*Sh*). Core subsets constructed by different sampling methods at 16% sample size. (1) When optimizing each of the four parameters independently; “*D_CE_”, “Sh”, “He”, “Cv* strategy*”.* (2) When a weight of 60% was assigned to *D_CE_* and 40% to Cv; “*D_CE_*Cv strategy”. (3) When optimizing all parameters simultaneously with equal weight given to each parameter; “multi-strategy”. Numbers in brackets and dotted lines indicate the number of alleles captured and the four allocation sampling strategies considered optimal, respectively.

This comparison was carried out using the 502 SSR profiles with a 16% sample size determined previously by *Mstrat*. All core sets sampled by different methods outperformed CC9-80 (core chosen randomly) in which the *D_CE_, He*, and *Sh* values were quite similar to those of OWGB Marrakech whereas the allelic richness values were significantly different from those of the whole collection (*p<0.05;* Table 1; Figure 2). When optimizing each of the four genetic parameters independently with the ASLS method, the sampled core subsets had the highest scores of all the core subsets with respect to the parameter being optimized, whereas other parameters not considered during optimization were highly affected (Table 1). For instance, with the “*D_CE_* strategy”, the selected core subset showed the highest *D_CE_* (CC2-80; 0.833±0.07**)**, while a low number of alleles was captured compared to the “*Cv* strategy” (only 234 among 279 alleles). For the MLST method, the CC8-80 core subset revealed higher *D_CE_* and similar *Sh* values as compared to CC6-60 and CC7-80, whereas fewer captured alleles were noted (only 236 alleles). Finally, four sampling strategies using the ASLS method showed better *D_CE_* and *Sh* scores than all other core subsets, including CC7-80, generated by the maximizing method (Table 1; Figure 2).

All methods allowed capture of at least 93.4% of the trait classes (CC9-80; Table 1) and all *cpDNA* haplotypes observed in OWGB Marrakech were captured in CC1-80 and CC7-80, whereas only 11 haplotypes (except E2-3 observed once for the “Lechin de Sevilla” variety from Spain) were captured when optimizing genetic parameters other than *Cv* using the ASLS method (Table 1).

According to the results, four allocation sampling strategies using the ASLS method were selected, i.e. “*D_CE_*”, “*He*”, “*Sh*”, and “multi-strategy” (Figure 2). Core subsets generated using the four strategies highlighted a trade-off in the genetic parameters considered in the study, including genetic distance (Table 1). These strategies were tested with different sample sizes (4, 8, 24, and 32%).

### Comparison of Sampling Size

**Figure 3:**
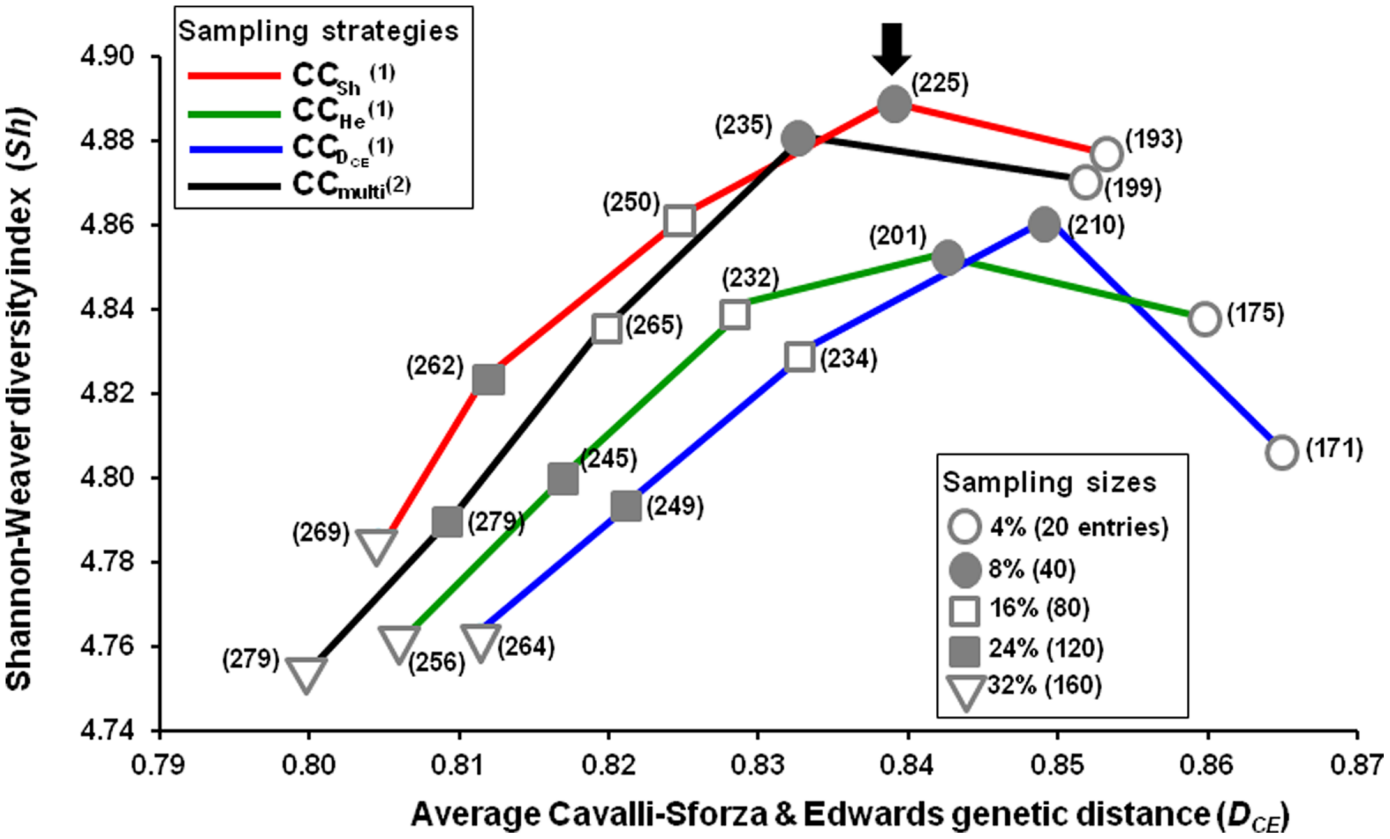
Comparison of sampling size according to average genetic distance (*D_CE_*) and Shannon-Weaver diversity index (*Sh*). Core subsets sampled at different sampling sizes using the four strategies of the ASLS method that was optimal at 16% sample size. (1) When optimizing each parameter independently; “*D_CE_”, “Sh”, and “He strategy”.* (2) When optimizing all parameters simultaneously with equal weight given to each parameter; “multi-strategy”. Numbers in brackets and arrows indicate the number of alleles captured and the chosen core subset as starting point for final core collections, respectively.

As shown in Figure 3, the sample size was inversely correlated with *D_CE_* and *Sh*, except for the 4% sample size, because of allelic redundancy within the core subset when the core size is increased. Increasing the sample size did not improve the capture of total alleles and trait classes, except for the “multi-strategy” where all alleles had been captured at 24% sample size (Table S3).

It would be unfeasible to design a core collection to fulfil all genetic measures at once because of the trade-off between genetic parameters. We thus propose a two-step method whereby one representative core subset of reasonable size is first selected, with a trade-off between *D_CE_*, *Sh*, *He*, and *Cv* genetic measures, and secondly a core subset is compiled with genotypes carrying missing alleles and trait classes. Hence, the CC2-40 core subset constructed using the “*Sh* strategy” with the *Core Hunter* program at 8% sample size was chosen as a starting point for the following steps since it nearly fulfilled all the required genetic parameters while being of suitable size (Table S3). However, eight among the 14 reference varieties defined above and two among the 12 haplotypes of OWGB Marrakech (E2.2 observed for “Trillo”, “Crastu”, and “Gremigno di Fuglia” varieties from Italy, and E2.3 for “Lechin de Sevilla” from Spain) were not captured in the CC2-40 core subset. When we examined alleles not captured in CC2-40 (54 among 279 alleles), it was found that 26 among the 54 alleles occurred once. Otherwise, all entries were conserved in successive constructed core subsets sampled by the “*Sh* strategy” while increasing the sample size, indicating the consistency of the sampling strategy and the robustness of the genetic parameter for selecting entries.

### Development of Final Core Collections

A primary core collection of 50 entries (CC_50_) was defined (Figure 1, step 1). This core collection includes the 40 entries of the CC2-40, “Lechin de sevilla” and “Trillo” varieties which each carry the two missing *cpDNA* haplotypes, and 8 missing reference varieties among the 14 defined above (Table S4; Figure S2, level 1). The 50 entries enabled capture of 229 alleles, 12 haplotypes, and 207 trait classes (Table 2) and reflected the geographic distribution of olive since varieties from 11 countries among 14 were represented (Table 3).

**Table 2:** Parameter measurements for different core collections and OWGB Marrakech.

	Size (%)	*Cv (%)*	*D_CE_ (±SD)*	*He*	*Sh*	# Trait classes (%)	# Haplotypes
**OWGB**	502	279 (100)	0.746 (±0.092)	0.728	4.524	213 (100)	12
**CC_50_**	50 (10)	229 (82)	0.812 (±0.074)	0.805*	4.825	207 (97.1)	12
**CC_92_**	92 (18.3)	279 (100)	0.785 (±0.074)	0.779	4.765	211 (99)	12
**CC_94_**	94 (18.7)	279 (100)	0.781 (±0.076)	0.777	4.75	213 (100)	12

*Cv:* allelic coverage or number of alleles, *D_CE_:* average genetic distance of Cavalli-Sforza and Edwards, *SD:* standard deviation, *He:* Nei diversity index, *Sh*: Shannon-Weaver diversity index.

* Statistically significant difference (p<0.05) using the Mann-Whitney test to assess differences between core collections and OWGB Marrakech.

**Table 3:** Number and frequency of genotypes per country in OWGB Marrakech and in both proposed core collections.

Geographical zone	Country	OWGB (%)^a^	CC_50_ (%)^b^	CC_94_ (%)^b^
West	Morocco	37 (7.4)	5 (13.5)	6 (16.2)
	Portugal	14 (2.8)	1 (7.1)	2 14.3)
	Spain	89 (17.7)	6 (6.7)	16 (18)
		140 (27.9)	12 (24)	24 (25.5)
Center	Algeria	38 (7.5)	4 (10.5)	5 (13.1)
	France	11 (2.2)	1 (9.1)	3 (27.2)
	Tunisia	23 (4.6)	3 (13)	4 (17.4)
	Italy	163 (32.4)	14 (8.6)	33 (20.2)
	Slovenia	9 (1.8)	–	–
	Croatia	14 (2.8)	–	2 (14.3)
	Greece	13 (2.6)	2 (15.4)	2 (15.4)
		271 (54)	24 (48)	49 (52.1)
East	Cyprus	16 (3.2)	1 (6.2)	1 (6.2)
	Egypt	19 (3.8)	4 (21)	5 (26.3)
	Lebanon	9 (1.8)	–	1 (11.1)
	Syria	47 (9.4)	9 (19.1)	14 (29.8)
		91 (18.1)	14 (28)	21 (22.4)
**Total**		**502**	**50 (10)** c	**94 (18.7)** c

The percentage of entries was calculated according to the number of available genotypes within each country.

a Frequency within OWGB Marrakech.

b Frequency proportional to the number of genotypes per country or geographical zone.

c Frequency proportional to the total number of genotypes within OWGB Marrakech.

Using the primary core collection (CC_50_) as a kernel (Figure 1, step 2), we estimated the minimum number of entries needed to capture all alleles and trait classes using the *Mstrat* program. The redundancy function of the program revealed that 94 entries (18.7%) were sufficient to capture the total diversity, i.e. allelic and phenotypic (Figure 1, step 2-A). Based on this sample size, 200 core collections were constructed with *Mstrat* (Table S4). For each core collection of 94 entries (CC_94_), 72 genotypes were found to be common in all of the 200 independent runs, i.e. the 50 genotypes used as a kernel and 22 genotypes carrying alleles observed once, while a combination of 22 complementary genotypes were selected among a panel of 106 genotypes shared between 200 runs (Figure 1; Figure S2, level 2). Arbitrarily selecting one core collection (CC1 in Table S4) revealed that all countries were represented, except for Slovenia which has 9 accessions in OWGB Marrakech (Table 3). Genotypes from this country were found in 73 of the 200 core collections (Table S4).

The effect of using phenotypic traits when sampling genotypes was tested by constructing core collections based only on nuclear data and CC_50_ as a kernel (Figure 1, step 2-B). The redundancy function of *Mstrat* program thus revealed that 92 entries (CC_92_) were necessary to capture all 279 alleles. As for CC_94_, 72 genotypes were common between all 200 constructed core collections of 92 entries (result not shown), whereas a panel of 91 genotypes could be used to select a combination of 20 complement genotypes to capture the total allelic diversity. One core collection of 92 entries among 200 was arbitrary chosen and compared to the above described CC_94_. The results indicated that 99% of the trait classes (211 among 213) were captured in this core collection and similar values were obtained regarding *D_CE_*, *Sh* and *He* for both core collections (Table 2). In addition, 85 genotypes were shared between CC_92_ and CC_94_. Hence, phenotypic data may have a limited effect since similar results were obtained regardless of the sampling method used, i.e. using trait classes or not.

### Genetic Structure and Representativeness of the Core Collections

**Figure 4:**
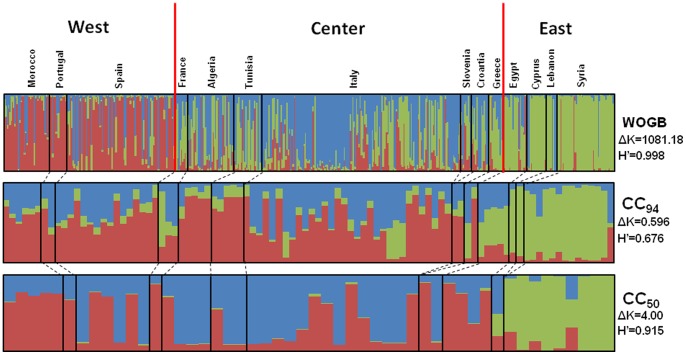
Inferred structure for K = 3 within OWGB Marrakech, CC_50_, and CC_94_. *H′* represents the similarity coefficient between runs, whereas ΔK represents the ad-hoc measure of Evanno et al. [84]. According to geographic and genetic criteria, three gene pools were revealed within Marrakech OWGB (western, central, and eastern Mediterranean groups) while the genetic structure was reduced to two sub-divisions in both core collections (eastern and western/central).

**Figure 5:**
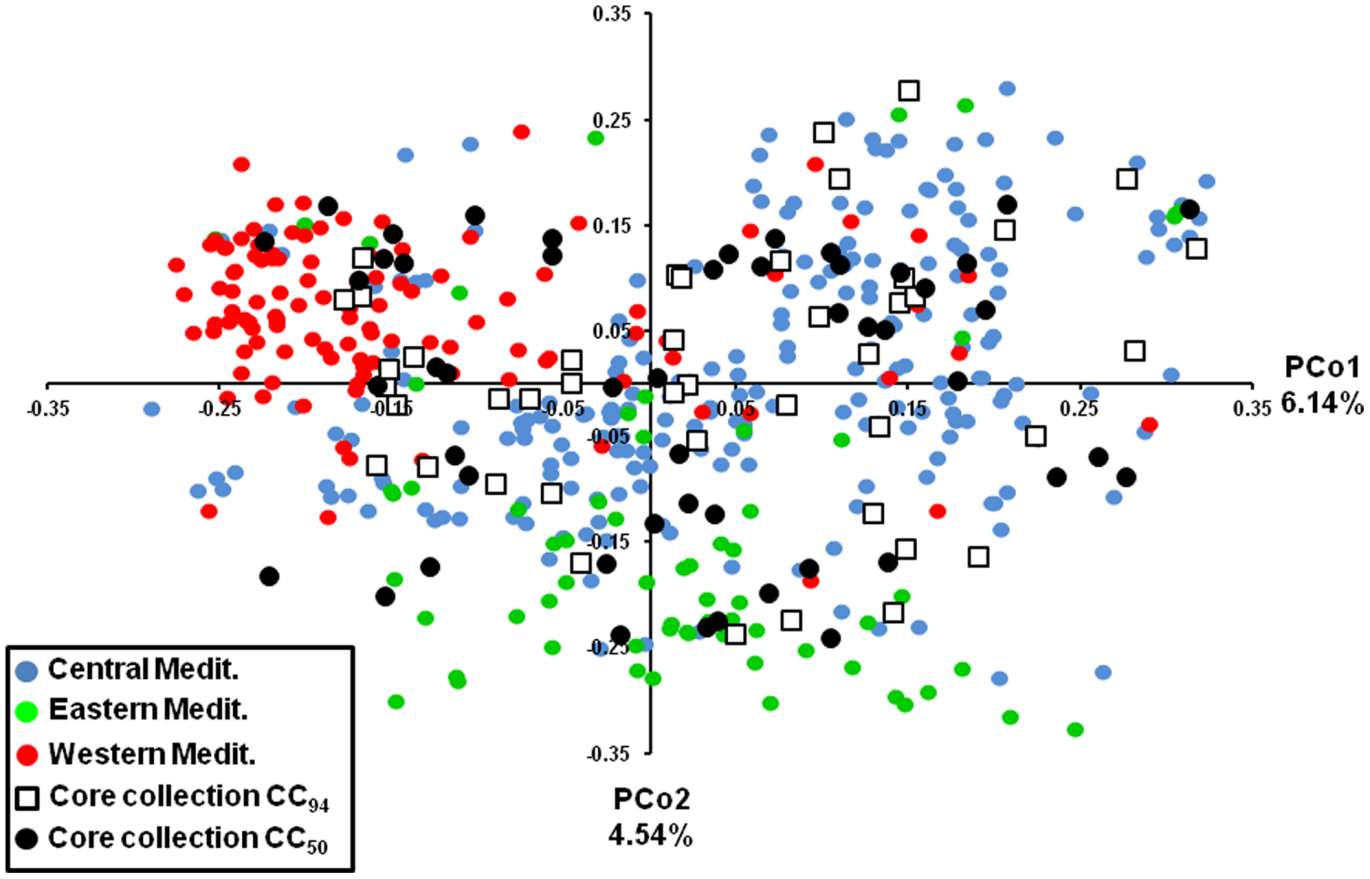
Two-dimensional distribution of the principal coordinate analysis (PCoA) for CC_50_, CC_94_ and OWGB Marrakech. Colours indicate the three gene pools (eastern, western and central Mediterranean Basin). The genetic variation of each principal coordinate (PCo1 and PCo2) is indicated. Both core subsets span the range of all genotypes among the three gene pools, whereas the majority of entries were found to occur in the central Mediterranean area.

Using model-based Bayesian clustering, the *Structure* program allowed classification of the 502 genotypes into three gene pools according to their regional origins (western, central, and eastern Mediterranean; Figure 4; Table S1), while the second most likely genetic structure was found at K = 5 (ΔK = 155.12 and *H′* = 0.992; Figure S3). Similar results were obtained when the analysis was conducted on genotypes distinguished by more than three dissimilar alleles (457 genotypes; results not shown). In both core collections (CC_50_ and CC_94_), the selected genotypes revealed a high level of admixture between gene pools. In fact, among the 50 and 94 genotypes, 23 (46%) and 71 (75.5%) were assigned to more than one gene pool with membership probabilities of less than 0.80, respectively. In addition, principal coordinate analysis (PCoA; Figure 5) revealed that both core collections encompassed the entire range of genotypes in the three gene pools, whereas 32 (64%) and 65 (69.1%) entries were classified into the central Mediterranean gene pool for the CC_50_ and CC_94_ core collections, respectively. Low *ΔK* and *H′* scores at K = 3 were noted for both core collections compared to OWGB Marrakech, therefore highlighting the absence of stability in obtaining runs at K = 3. Although high ΔK and *H′* scores at K = 5 were obtained for both core collections (Figure S3), no consistency in genetic structure was noted when plotting the Q scores (Figure S4), while the model at K = 3 indicated two subgroups for both CC_50_ and CC_94_; the first one contained entries originating from the western and central Mediterranean whereas the second included eastern Mediterranean varieties (Figure 4).

**Figure 6:**
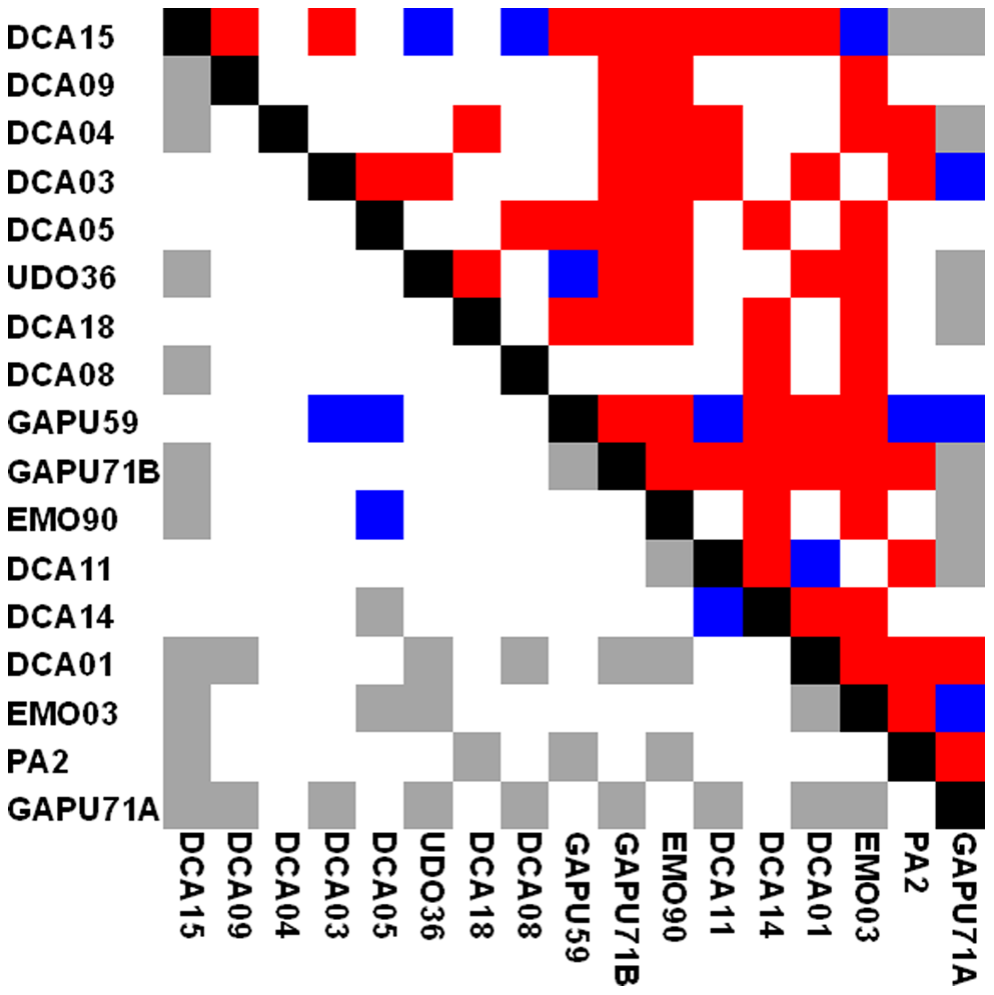
Linkage disequilibrium *p*-values between pairs of 17 SSR loci. Linkage disequilibrium *p-values* obtained for the 457 genotypes (distinguished by more than three dissimilar alleles, upper triangle) and for the CC_94_ core collection (lower triangle) using the *Tassel* program. Red, blue, grey and white boxes indicate high (p<0.0001), intermediate (0.01>p>0.0001), low significance (p>0.01) and no significance, respectively. A sampling effect on the linkage disequilibrium was found between pairs of SSR loci.

**Figure 7:**
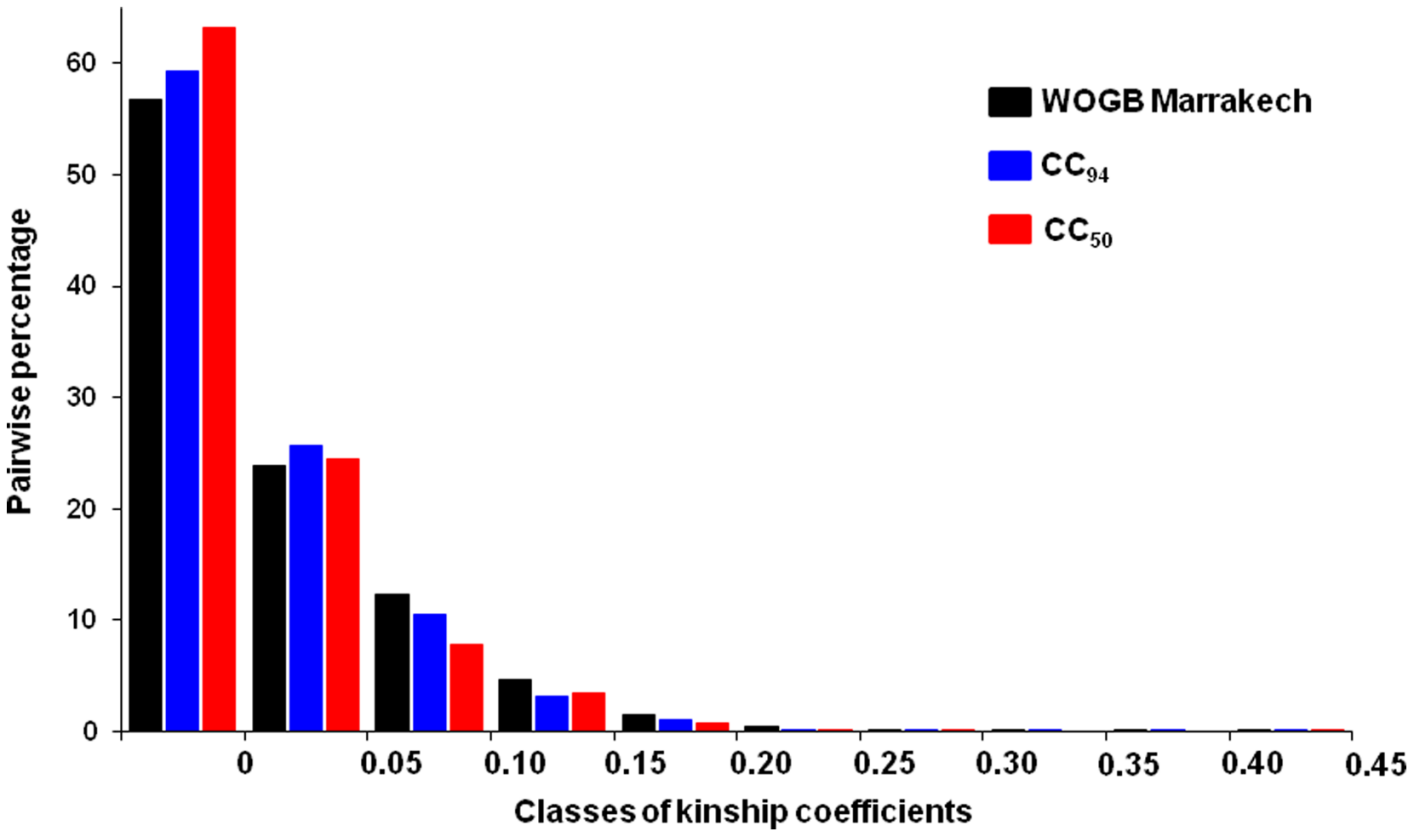
Frequency distribution of the pairwise relative kinship coefficient. Pairwise relative kinship coefficient for the 457 genotypes of OWGB Marrakech, CC_50,_ and CC_94_ using 17 SSR loci. Values equal to or greater than 0.45 were grouped as 0.45. The kinship calculation indicated a low level of relatedness between genotypes, with only a few genotypes being more related to each other.

When considering only 457 genotypes distinguished by more than three dissimilar alleles, the LD scores (*r^2^*) were significant for 59.5% of the pairwise comparisons (81 among 136 pairwise comparisons), while only 26.5% of the pairwise comparisons displayed a significant LD in CC_94_ (Figure 6). The relative kinship computed for both core collections showed a high pairwise frequency at 0–0.05 (87.6% for CC_50_ and 84.9% for CC_94_), whereas it decreased progressively between 0.05 and 0.45 (7.8% and 10.4% to 0.08% and 0.04% for CC_50_ and CC_94_, respectively; Figure 7).

## Discussion

The aim of the study was to construct flexible core collections for cultivated olive, of a manageable working size for conducting association mapping studies, by sampling the minimum number of entries that maximize the representativeness of allelic and phenotypic diversity. Such working core collections facilitate experimental trials to assess germplasm under contrasting environmental conditions. We analyzed our results with regards to: (1) the representativeness of the Marrakech OWGB, (2) tools and criteria used for defining the core collections, and (3) the efficiency of the developed core collections for genetic association mapping.

### OWGB Marrakech is Representative of Mediterranean Olive Diversity

Despite the presence of similar proportions of alleles with frequencies <1% and those observed only once in both OWGB collections (Text S2; 53.4% and 19.5% in OWGB Cordoba, respectively) [66], a higher allelic richness was noted in OWGB Marrakech than in OWGB Cordoba (16.41 and 11.38 alleles/locus [51], respectively). OWGB Marrakech was found to be more diversified than OWGB Cordoba as shown by the presence of more accessions from different countries, particularly those from the eastern Mediterranean [52]. OWGB Marrakech has more Egyptian (19 genotypes), Syrian (47), and Lebanese (9) genotypes than OWGB Cordoba, while more than 55% of all accessions in OWGB Cordoba are from Spain [51], [66]. The entire diversity observed in OWGB Marrakech is explained mainly by the scientific context when setting up the collection. The germplasm bank was set up with previously characterized genetic resources, including agro-morphological descriptors and/or molecular markers from each Mediterranean country, in order to optimize the available olive germplasm [52]. The olive germplasm available in OWGB Marrakech better reflects the genetic structure of cultivated olive in the Mediterranean basin, since three gene pools were distinguished, i.e. western, eastern and central Mediterranean, as also reported by Sarri et al. [57] and Baldoni et al. [58] using different sets of SSR markers, while only two were revealed in OWGB Cordoba by Belaj et al. [51], i.e. western and eastern/central Mediterranean. Therefore, we consider that OWGB Marrakech is particularly suitable for association mapping studies and also for establishing representative core collections since it encompasses a high range of olive germplasm from the Mediterranean Basin, including the eastern gene pool. Nevertheless, a simultaneous analysis of both germplasm banks, as one single dataset, with the same set of molecular markers to construct a real core collection representing Mediterranean olive germplasm will certainly provide complementary information and thus be an asset for olive genetic research.

### Effectiveness of Processed Data in Constructing Core Collections

Accessions with similar phenotypes may not necessarily have a close genetic relationship [38] because of the polygenic properties of most traits and the effect of the environment on the expression of the trait being analyzed. Hence, applying molecular marker information reflecting the DNA polymorphism pattern is a powerful tool in core collection development. The cost, time, and effort required for phenotypic characterization, especially in a woody perennial species collection, are much greater than required for an assessment using molecular tools. As most of current 17 loci are well-scattered throughout linkage groups [89]–[90], we assume that the applied set of SSRs may be effective to obtain an overview of olive diversity as observed in other studies [29], [36]. Further studies using other sets of molecular markers (e.g. SNP) could confirm our assumption. Furthermore, despite the fact that maternal lineage polymorphism of is lower within olive varieties than noted in olive oleasters [67], therefore chloroplast sequence information is substantial when establishing core collections. This information optimises sampling to clarify the evolutionary history of olive varieties and therefore their involvement in agronomic traits of interest alone or in association with nuclear genes.

Otherwise, the compiled phenotypic data was used with caution in the present study since not all varieties were completely characterized with the 72 agro-morphological traits and phenotypic data was gathered from different olive databases according to the variety names [68]–[72]. As we could not exclude the presence of distinct genotypes with the same name due to mislabeling and synonymy cases [55], such data could be useful to conduct a first screening on phenotypic variability of olive varieties in OWGB Marrakech. Their use could provide additional and qualitative information to choose entries covering the range of variability of phenotypic traits. Whatever their level of representativeness of phenotypic variability in Mediterranean olive, these traits may have a limited effect on the sampling entries since we obtained similar results using phenotypic trait classes or not. Further field assessments are clearly required to obtain more reliable and comprehensive data on the phenotypic diversity of selected entries.

### Core Collections are Highly Representative of the Overall Olive Genetic Variability

The broad diversity in the Marrakech OWGB could be represented in two core collections of 50 (10%) and 94 (18.7%) entries capturing 82 and 100% of the total allelic diversity, respectively. A decrease in *D_CE_*, *He*, and *Sh* scores was noted when the core collection size was increased from 50 to 94 entries (Table 2). This could mainly be explained by the redundancy of the information provided by each additional genotype, since the entries added to the initial 50 genotypes contributed less than two alleles each, i.e. 44 added entries provided only 50 additional alleles (mean of 1.13 alleles/entry). A size of 94 entries, capturing the total diversity, is suitable for field assessments with many replicates for association mapping since many studies have been conducted on annual and perennial species represented by a similar number of accessions characterized by high genetic diversity in their original collections: 95 accessions for *Triticum aestivum* [19]; 96 for *Arabidopsis thaliana* [91] and *Lolium perenne* [92]; and 104 for *Prunus persica* [27].

Taking into account the trade-off between genetic parameters, we consider that the two-step method is a suitable to overcome these constraints and it could be applied to other annual and perennial species. The Shannon-Weaver diversity index was shown to be an adequate first criterion to be optimized to select core subsets with optimal allelic coverage and genetic distance. Basically, the index accounts for the allelic richness (number of distinct alleles) and the evenness (distribution of different alleles) within a given sample [43]. The Shannon-Weaver diversity index can be used for sampling individuals to capture the most allelic variation while eliminating those containing the most-represented alleles, i.e. all alleles are equally represented. To our knowledge, it is the first attempt to use the Shannon-Weaver diversity index as a first criterion to set up core collections, whereas it has been frequently used in other studies to validate the relevance of constructed core subsets [29]–[30], [79], [93]. This genetic parameter could be used as a first criterion to enhance field experimentation since it reduces artefacts resulting from the dominance of some categories (alleles and/or trait classes) over others.

Both core collections (CC_50_ and CC_94_) are of reasonable size as previous studies proposed 5–20% core sizes, capturing at least 70% of the genetic diversity [46]. CC_94_ is similar in size to core collections previously obtained in *Olea europaea* [51], [66] and *Pyrus communis* [94]. However, as compared to other perennial and highly heterozygotic species, this sample size is considered to be higher than those obtained in *Annona cherimola* (14.3%, 40 entries) [33], *Malus sieversii* (10.5%, 84) [34] and *Vitis vinifera* (4%, 92) [36]. This may be explained by the high diversity and the low redundancy in Marrakech OWGB as compared to the high redundancy and presence of many accessions of clonal origin in the *Vitis* collection [95].

By contrast to previously developed olive core collections, the proposed two-step method may be used to develop many core collections with one common set of 72 varieties and 22 different varieties. In fact, CC_94_ is a flexible core collection in which 200 specific combinations of 22 varieties are available that can be chosen on the basis of many criteria, such as; geographic origin, economic importance, traits of interest, and/or previous use in breeding programs. This approach enables experimental flexibility and rational choice of varieties to be studied, with the possibility of adding supplementary genotypes to the initial core collection of 94 entries, if necessary.

Despite using different sampling algorithms, Belaj et al. [51] and Diez et al. [66] proposed core collections by maximizing only the number of alleles as the main criterion. Here we were able to construct core collections by taking many criteria at once into account, including sampling of genetically distant varieties. Moreover, a substantial over-representation of western accessions was noted in both previous olive core collections, since 46% of the entries originated from the western Mediterranean gene pool, mainly from Spain, versus 30% and 24% from eastern and central gene pools, respectively. By contrast, both core collections proposed in the current study accurately reflected the geographic distribution of cultivated olive, and demonstrated the high admixture level, since 48% and 52% of 50 and 94 entries, respectively, originated from the central Mediterranean zone. Our proposal is supported by the fact that the central Mediterranean zone is a hybrid area between the eastern and western zones, as shown by the admixed inferred ancestry of most of the genotypes sampled in this area [52], [96]. Strikingly, when comparing the varietal composition in the CC_94_ core collection with those previously published for olive, we found that only 11 and 12 varieties were shared with those reported by Diez et al. [66] and Belaj et al. [51], respectively. This finding could mainly be explained by the different sampling approaches used to construct core collections and by the differences in the original OWGB collections regarding the genetic diversity and varietal composition, since only 153 varieties are common to both OWGB collections [52].

### Core Collections are Promising for Association Mapping

Unidentified population sub-divisions that have occurred through the evolutionary history of species (bottleneck effect, domestication processes), local adaptation and/or selection, is a major constraint for association mapping because of the many false positives that occur [23], [80]–[82]. Hence, information on genetic structures, the extent of LD and the relatedness between genotypes is crucial for association mapping. Ideally, samples should have a minimal population structure or familial relatedness to achieve the best statistical power [80]. Here we considered two sub-divisions within the proposed core collections depicting the genetic structure of OWGB Marrakech classified into three gene pools. In addition, there was evidence of spurious LD between unlinked SSR loci in nearly all of the pairwise tests in the whole collection (Figure 6). This could mainly be explained by the genetic sub-division within OWGB Marrakech, as noted by the model-based Bayesian clustering, whereas a contrasting change in LD measurements was noted in the CC_94_ core collection. As reported by Breseghello and Sorrells [19] and Pessoa-Filho et al. [44], the significant reduction in spurious disequilibrium is mainly due to sampling effects when diversity was maximized, while the spurious LD that remained in the CC_94_ core collection was possibly caused by the low genetic structure in the 94 sampled entries. The assessment of relative kinship showed that most genotypes in OWGB Marrakech were significantly unrelated (80.6% of pairwise comparisons at 0–0.05). Similar genotype relatedness patterns were noted in both core collections (87.6 and 84.9% for CC_50_ and CC_94_, respectively). Our findings were similar to those obtained in *Brassica napus* [97], *B. rapa* [98], and *Zea mays* [12] for which relative kinship estimates indicated a low level of relatedness between genotypes, with only a few pairs of genotypes being more related than any pair taken at random in the selected sub-sample. Basically, since a set of unrelated individuals displays variation in many phenotypic traits, many association traits/markers can be studied in the same panel of individuals [80]. The proposed core collections are relevant for genetic association studies because of the genetic structures and relatedness [15], [97]. These could be included as co-variance parameters in models to control false positive markers-traits in association mapping analyses [23], [80]–[82].

### Conclusion

Our two-step method was shown to be well-adapted for constructing core collections of a size suitable for transfer within the scientific community. Such core collections are suitable for association mapping as they accommodate many genetic criteria and provide potential users with more flexibility for choosing varieties. It has been demonstrated that both proposed core collections clearly reflected the geographic and genetic diversity of olive, so they will be of major interest for breeding researchers to help them conduct comparative trails.

This work represents a preliminary step towards developing association mapping studies by sampling core collections and assessing the structure and relatedness within samples. Note that the proposed core collections should be periodically updated by including additional olive germplasm in the base collection and adding novel molecular markers such as SNPs. At the current state, the developed core collections will be useful for conducting field assessments and suitable for developing a long-term strategy for genome-wide association studies in olive.

## Supporting Information

Figure S1 **Maximizing average Cavalli-Sforza & Edwards genetic distance (** ***D_CE_*** **) and allelic coverage (** ***Cv*** **).** Values of *D_CE_* and *Cv* were maximized simultaneously with respect to a weight assigned to each measure. The *Core Hunter* program was run independently for 10 different weight values assigned to *D_CE_* and *Cv measures*; (1) When a weight of 100% was assigned to *Cv*, (2) when a weight of 40% was assigned to *Cv* and 60% to *D_CE_*, and (3) when a weight of 100% was assigned to *D_CE_*.
(TIF)

Figure S2 **Three different levels proposed for core collections.** Level 1 (L1) represents the primary core collection (CC_50_), which includes the 40 entries selected using the “Sh strategy” implemented in *Core Hunter* program at 8%, two varieties carrying the two missing *cpDNA* haplotypes, and 8 non-selected reference varieties among the 14. Level 2 includes accessions carrying alleles observed once (22 genotypes). Level 3 represents final core collections (CC_94_) constructed by adding a complement of 22 genotypes to the previous 72 among a panel of 106 genotypes to capture the total allelic and phenotypic diversity.
(TIF)

Figure S3 **Plot of ad-hoc ΔK measurements and coefficients of similarity (** ***H′*** **) for K between 2 and 7.** Arrows indicate the best genetic structure model for both core collections and OWGB Marrakech. According to both parameters, i.e. *ΔK* and *H′*, the best genetic structure model was not stable, while it is defined at K = 3 in Marrakech OWGB, indicating the absence of an obvious genetic structure in the core collections (see Figure S3).
(TIF)

Figure S4 **Inferred structure for K = 5 clusters within OWGB Marrakech, CC_50_, and CC_94_ core collections.** *H′* represents the similarity coefficient between runs, and ΔK represents the ad-hoc measure of Evanno et al. [84]. No consistency was observed in genetic structures based on more than three clusters.
(TIF)

Table S1 **List of 502 genotypes used in the present study classified according to distinct genotypes (SSR profiles), origin, maternal lineage and inferred ancestry (Q matrix) at K = 3 clusters.**
(XLS)

Table S2 **List of traits, number of trait classes according to standards described by the International Olive Oil Council, and number of varieties with available phenotypic data.** The number of varieties differed according to traits indicates that there was missing data, and that not all varieties were completely characterized with the 72 phenotypic traits.
(DOC)

Table S3 **Genetic parameters of core subsets sampled using four different strategies with the ASLS method at four sample sizes, i.e. 4, 8, 24, and 32%.** The CC2-40 core subset (in bold) was chosen as the optimal to construct final core collections.
(DOC)

Table S4 **List of 200 core collections with a 94 sample size (CC_94_) generated with** ***Mstrat*** **using the core collection of 50 entries as a kernel (CC_50_).** (x) Corresponds to the presence of the accession in the core collection concerned. The CC level column indicates the level of the core collection as shown in Figure S2. No differences between the 200 cores were observed for the Nei diversity index.
(XLS)

Text S1 **Protocols of nuclear and chloroplast loci analyses.**
(DOC)

Text S2 **Genetic analysis of OWGB Marrakech.**
(DOC)

## References

[1] The Arabidopsis Genome Initiative (2000) Analysis of the genome sequence of the flowering plant *Arabidopsis thaliana* . Nature 408: 796–815. 11130711 10.1038/35048692

[2] Varshney RK, Nayak SN, May GD, Jackson SA (2009) Next generation sequencing technologies and their implications for crop genetics and breeding. Trends Biotechnol 27: 522–530. 19679362 10.1016/j.tibtech.2009.05.006

[3] Heffner EL, Sorrells ME, Jannink JL (2009) Genomic selection for crop improvement. Crop Sci 49: 1–12.

[4] Jannink JL, Lorenz AJ, Iwata H (2010) Genomic selection in plant breeding: from theory to practice. Brief Funct Genomics 9: 166–177. 20156985 10.1093/bfgp/elq001

[5] Mackay I, Powell W (2007) Methods for linkage disequilibrium mapping in crops. Trends Plant Sci 12: 57–63. 17224302 10.1016/j.tplants.2006.12.001

[6] Collard BCY, Jahufer MZZ, Brouwer JB, Pang ECK (2005) An introduction to markers, quantitative trait loci (QTL) mapping and marker-assisted selection for crop improvement: The basic concepts. Euphytica 142: 169–196.

[7] Weiss KM, Clark AG (2002) Linkage disequilibrium and the mapping of complex human traits. Trends Genet 18: 19–24. 11750696 10.1016/s0168-9525(01)02550-1

[8] Myles S, Peiffer J, Brown PJ, Ersoz ES, Zhang Z, et al (2009) Association mapping: Critical considerations shift from genotyping to experimental design. The Plant Cell 21: 2194–2202. 19654263 10.1105/tpc.109.068437 PMC2751942

[9] Rafalski JA (2010) Association genetics in crop improvement. Curr Opin Plant Biol 13: 174–180. 20089441 10.1016/j.pbi.2009.12.004

[10] Abdurakhmonov IY, Abdukarimov A (2008) Application of association mapping to understanding the genetic diversity of plant germplasm resources. Int J Plant Genomics. Doi: 10.1155/2008/574927. 10.1155/2008/574927 PMC2423417 18551188

[11] Barnaud AA, Lacombe TT, Doligez AA (2006) Linkage disequilibrium in cultivated grapevine, *Vitis vinifera* L. Theor Appl Genet. 112: 708–716. 10.1007/s00122-005-0174-1 16402190

[12] Yan J, Shah T, Warburton ML, Buckler ES, McMullen MD, et al (2009) Genetic characterization and linkage disequilibrium estimation of a global maize collection using SNP markers. PloS ONE 4: e8451. 20041112 10.1371/journal.pone.0008451 PMC2795174

[13] Aranzana MJ, Abbassi EK, Howad W, Arus P (2010) Genetic variation, population structure and linkage disequilibrium in peach commercial varieties. BMC Genetics 11: 69. 20646280 10.1186/1471-2156-11-69 PMC2915947

[14] Arunyawat U, Capdeville G, Decroocq V, Mariette S (2012) Linkage disequilibrium in Frensh wild cherry germplasm and worldwide sweet cherry germplasm. Tree Genet Genomes. doi: 10.1007/s11295-011-0460-9.

[15] Aranzana MJ, Kim S, Zhao K, Bakker E, Horton M, et al (2005) Genome-wide association mapping in *Arabidopsis* identifies previously known flowering time and pathogen resistance genes. PLoS Genet 1(5): e60 doi:10.1371/journal.pgen.0010060. 16292355 10.1371/journal.pgen.0010060 PMC1283159

[16] Brachi B, Faure N, Horton M, Flahauw E, Vazquez A, et al (2010) Linkage and association mapping of *Arabidopsis thaliana* flowering time in nature. Plos Genetics 6(5): e1000940. 20463887 10.1371/journal.pgen.1000940 PMC2865524

[17] Agrama HA, Eizenga GC, Yan W (2007) Association mapping of yield and its components in rice cultivars. Mol Breed 19: 341–356.

[18] De Oliveira Borba TC, Brondani RP, Breseghello F, Coelho AS, Mendonça JA, et al (2010) Association mapping for yield and grain quality traits in rice (*Oryza sativa* L.). Genet Mol Biol. 33: 515–24. 10.1590/S1415-47572010005000065 PMC3036121 21637426

[19] Breseghello F, Sorrells ME (2006) Association mapping of kernel size and milling quality in wheat (*Triticum aestivum* L.) cultivars. Genetics 172: 1165–1177. 16079235 10.1534/genetics.105.044586 PMC1456215

[20] Thornsberry JM, Goodman MM, Doebley J, Kresovich S, Nielsen D, et al (2001) Dwarf8 polymorphisms associate with variation in flowering time. Nature genetics 28: 286–289. 11431702 10.1038/90135

[21] Remington DL, Thornsberry JM, Matsuoka Y, Wilson LM, Whitt SR, et al (2001) Structure of linkage disequilibrium and phenotypic associations in the maize genome. Proc Natl Acad Sci U S A 98: 11479–84. 11562485 10.1073/pnas.201394398 PMC58755

[22] Blair MW, Díaz LM, Buendía HF, Duque MC (2009) Genetic diversity, seed size associations and population structure of a core collection of common beans (*Phaseolus vulgaris* L.). Theor Appl Genet 119: 955–972. 19688198 10.1007/s00122-009-1064-8

[23] Malosetti M, Van der Linden CG, Vosman B, Van Eeuwijk FA (2007) A mixed-model approach to association mapping using pedigree information with an illustration of resistance to *phytophthora infestans* in potato. Genetics 175: 879–889. 17151263 10.1534/genetics.105.054932 PMC1800631

[24] Wei XM, Jackson PA, McIntyre CL, Aitken KS, Croft B (2006) Associations between DNA markers and resistance to diseases in sugarcane and effects of population substructure. Theor Appl Genet 114: 155–164. 17047910 10.1007/s00122-006-0418-8

[25] Gonzalez-Martinez SC, Ersoz E, Brown GR, Wheeler NC, Neale DB (2006) DNA sequence variation and selection of Tag single-nucleotide polymorphisms at candidate genes for drought-stress response in *Pinus taeda* L. Genetics. 172: 1915–1926. 10.1534/genetics.105.047126 PMC1456261 16387885

[26] Thumma BR, Nolan MF (2005) Polymorphisms in cinnamoyl CoA reductase (CCR) are associated with the variation in microfibril angle in *Eucalyptus* spp. Genetics 173: 1257–1265. 10.1534/genetics.105.042028 PMC1456829 16085705

[27] Cao K, Wang L, Zhu G, Fang W, Chen C, et al. (2012) Genetic diversity, linkage disequilibrium, and association mapping and analyses of peach (*Prunus persica*) landraces in China. Tree Genet Genomes. doi:10.1007/s11295-012-0477-8.

[28] Frankel OH, Brown AHD (1984) Plant genetic resources today: a critical appraisal. In crop genetic resources: conservation and evaluation (Holden JHW and Williams JT. eds). London. 249–257.

[29] McKhann HI, Camilleri C, Berard A, Bataillon T, David JL, et al (2004) Nested core collections maximizing genetic diversity in *Arabidopsis thaliana* . Plant J 38: 193–202. 15053772 10.1111/j.1365-313X.2004.02034.x

[30] Zhao W, Cho GT, Ma KH, Chung JW, Gwag JG, et al (2010) Development of an allele-mining set in rice using a heuristic algorithm and SSR genotype data with least redundancy for the post-genomic era. Mol Breeding 26: 639–651.

[31] Balfourier F, Roussel V, Strelchenko P, Exbrayat-Vinson F, Sourdille P, et al (2007) A worldwide bread wheat core collection arrayed in a 384-well plate. Theor Appl Genet 114: 1265–1275. 17318494 10.1007/s00122-007-0517-1

[32] Franco J, Crossa J, Taba S, Shands H (2005) A sampling strategy for conserving genetic diversity when forming core subsets. Crop Sci 45: 1035–1044.

[33] Escribano P, Viruel MA, Hormaza JI (2008) Comparison of different methods to construct a core germplasm collection in woody perennial species with simple sequence repeat markers. A case study in cherimoya (*Annona cherimola*, Annonaceae), an underutilised subtropical fruit tree species. Ann Appl Biol 153: 25–32.

[34] Richards CM, Volk GM, Reeves PA, Reilley AA, Henk AD (2009) Selection of stratified core sets representing wild Apple (*Malus sieversii*). J Am Soc Hortic Sci 134: 228–235.

[35] Wang Y, Zhang J, Sun H, Ning N, Yang L (2011) Construction and evaluation of a primary core collection of apricot germplasm in China. Sci Hortic-Amsterdam 128: 311–319.

[36] Le Cunff L, Fournier-Level A, Laucou V, Vezzulli S, Lacombe T, et al (2008) Construction of nested genetic core collections to optimize the exploitation of natural diversity in *Vitis vinifera* L. subsp. Sativa. BMC Plant Biology 8: 31. 18384667 10.1186/1471-2229-8-31 PMC2375891

[37] Schoen DJ, Brown AHD (1993) Conservation of allelic richness in wild crop relatives is aided by assessment of genetic markers. Proc Natl Acad Sci U S A 38: 10623–10627. 10.1073/pnas.90.22.10623 PMC47829 8248153

[38] Marita JM, Rodriguez JM, Nienhuis J (2000) Development of an algorithm identifying maximally diverse core collections. Genet Resour Crop Evol 47: 515–526.

[39] Hu J, Zhu J, Xu HM (2000) Methods of constructing core collections by stepwise clustering with three sampling strategies based on the genotypic values of crops. Theor Appl Genet 101: 264–268.

[40] Gouesnard B, Bataillon TM, Decoux G, Rozale C, Schoen DJ, et al (2001) *Mstrat*: An algorithm for building germplasm core collections by maximizing allelic or phenotypic richness. J Hered 92: 93–94. 11336240 10.1093/jhered/92.1.93

[41] Perrier X, Flori A, Bonnot F (2003) Data analysis methods. In: Hamon, P, Seguin, M, Perrier, X,Glaszmann, J C. Ed., Genetic diversity of cultivated tropical plants. Enfield, Science Publishers. Montpellier. 43–76.

[42] Franco J, Crossa J, Warburton ML, Taba S (2006) Sampling strategies for conserving maize diversity when forming core subsets using genetic markers. Crop sci 46: 854–864.

[43] Thachuk C, Crossa J, Franco J, Dreisigacker S, Warburton M, et al (2009) *Core Hunter*: an algorithm for sampling genetic resources based on multiple genetics measures. Bioinformatics 10: 243. 19660135 10.1186/1471-2105-10-243 PMC2734557

[44] Pessoa-Filho M, Rangel PHN, Ferreira ME (2010) Extracting samples of high diversity from thematic collections of large gene banks using a genetic-distance based approach. BMC Plant Biol 10: 127. 20576152 10.1186/1471-2229-10-127 PMC3095284

[45] Brown ADH (1989) Core collections: A practical approach to genetic resources management. Genome 31: 818–824.

[46] Van Hintum TJL, Brown AHD, Spillane C, Hodgkin T (2000) Core collections of plant genetic resources. IPGRI Technical Bulletin No.3. International Plant Genetic Resources, Rome, Italy.

[47] Zohary D, Hopf M (2000) Domestication of plants in the old world: the origin and spread of cultivated plants in West Asia, Europe, and the Nile Valley. Oxford University Press, New York.

[48] Bartolini G, Prevost G, Messeri C, Carignani G (1999) Olive cultivar names and synonyms and collections detected in a literature review. Acta Hortic 474: 159–162.

[49] Bartolini G, Petruccelli R (2002) Classification, origin, diffusion and history of the olive. Plant Production and Protection Div, FAO, Rome (Italy). pp: 85.

[50] Bartolini G, Prevost G, Messeri C, Carignani C (2005) Olive germplasm: cultivars and world-wide collections. FAO/Plant Production and Protection, Rome. Available: http://www.oleadb.it. Accessed 2012 April 15.

[51] Belaj A, Dominguez-García MC, Atienza SC, Urdíroz NM, De la Rosa R, et al (2012) Developing a core collection of olive (*Olea europaea* L.) based on molecular markers (DArTs, SSRs, SNPs) and agronomic traits. Tree Genet Genomes 8: 365–378.

[52] Haouane H, El Bakkali A, Moukhli A, Tollon C, Santoni S, et al (2011) Genetic structure and core collection of the World Olive Germplasm Bank of Marrakech: towards the optimised management and use of Mediterranean olive genetic resources. Genetica 139: 1083–1094. 21960415 10.1007/s10709-011-9608-7 PMC3247671

[53] Angiolillo A, Mencuccini L, Baldoni L (1999) Olive genetic diversity assessed using Amplified Fragment Lenght Polymorphism. Theor Appl Genet 98: 411–421.

[54] Besnard G, Breton C, Baradat P, Khadari B, Bervillé A (2001) Cultivar identification in the olive (Olea europaea L.) based on RAPDS. J Am Soc Hortic Sci 126: 668–675.

[55] Khadari B, Breton C, Moutier N, Roger JP, Besnard G, et al (2003) The use of molecular markers for germplasm management in French olive collection. Theor Appl Genet 106: 521–529. 12589553 10.1007/s00122-002-1079-x

[56] Reale S, Doveri S, Díaz A, Angiolillo A, Lucentini L, et al (2006) SNP-based markers for discriminating olive (*Olea europaea* L.) cultivars. Genome 49: 1193–205. 17110999 10.1139/g06-068

[57] Sarri V, Baldoni L, Porceddu A, Cultrera NGM, Contento A, et al (2006) Microsatellite markers are powerful tools for discriminating among olive cultivars and assigning them to geographically defined populations. Genome 49: 1606–1615. 17426775 10.1139/g06-126

[58] Baldoni L, Cultrera NG, Mariotti R, Ricciolini C, Arcioni S, et al (2009) A consensus list of microsatellite markers for olive genotyping. Mol Breed 24: 213–231.

[59] Bellini E, Giordani E, Rosati A (2008) Genetic improvement of olive from clonal selection to cross-breeding programs. Adv Hortic Sci 22: 73–86.

[60] Santos-Antunes AF, Mohedo A, Trujillo I, Rallo L (1999) Influence of the genitors on the flowering of olive seedlings under forced growth. Acta Hortic 474: 103–105.

[61] Rosati A, Zipanćič M, Caporali S, Paoletti A (2010) Fruit set is inversely related to flower and fruit weight in olive (*Olea europaea* L.). Sci Hortic-Amsterdam126: 200–204.

[62] Prista T, Voyiatzi C, Metaxas D, Voyiatzis D, Koutsika Sotiriou M (1999) Observations on germination capacity and breeding value of seedlings of some olive cultivars. Acta Hortic 474: 117–120.

[63] Padula G, Giordani E, Bellini E, Rosati A, Pandolfi S, et al (2008) Field evaluation of new olive (*Olea europaea* L.) selections and effects of genotype and environment on productivity and fruit characteristics. Adv Hortic Sci 22: 87–94.

[64] Ripa V, De Rose F, Caravita MA, Parise MR, Perri E, et al (2008) Qualitative evaluation of olive oils from new olive selections and effects of genotype and environment on oil quality. Adv Hortic Sci 22: 95–103.

[65] Ben Sadok I, Moutier N, Garcia G, Dosba F, Grati-Kamoun N, et al.. (2012) Genetic determinism of the vegetative and reproductive traits in a F1 olive tree progeny: evidence of the tree ontogeny effect. Tree Genet Genomes. doi: 10.1007/s11295-012-0548-x.

[66] Díez CM, Imperato A, Rallo L, Barranco D, Trujillo I (2012) Worldwide core collection of olive cultivars based on Simple Sequence Repeat and morphological markers. Crop Sci 52: 211–221.

[67] Besnard G, Hernandez P, Khadari B, Dorado G, Savolainen V (2011) Genomic profiling of plastid DNA variation in the Mediterranean olive tree. BMC Plant Biol 11: 80. 21569271 10.1186/1471-2229-11-80 PMC3115843

[68] Bartolini G, Prevost G, Messeri C, Carignani G (1998) Olive germplasm: Cultivars and world-wide collections. FAO Library. Rome, Italy.

[69] Bartolini G (2008) Olive germplasm (Olea europaea L.), cultivars, synonyms, cultivation area, collections, descriptors. Available: http://www.oleadb.it/olivodb.html. Accessed 2012 May 6.

[70] Trigui A, Msallem M (2002) Oliviers de Tunisie: catalogue des variétés autochtones et types locaux. Volume I (Identification variétale and caractérisation morpho-pomologique des ressources génétiques oléicoles de Tunisie). Tunis, Tunisie: IRESA press. 159 p.

[71] Moutier N, Artaud J, Burgevin JF, Khadari B, Martre A, et al.. (2004) Identification et caractérisation des variétés d’olivier cultivées en France. Tome 1. Turriers: Naturalia publications. 245 p.

[72] Mendil M, Sebai A (2006) Catalogue des Variété Algériennes de l’Olivier. Ministère de l’agriculture et du développement rural, ITAF Alger, Algeria. 98 p.

[73] Shannon CE, Weaver W (1949) The Mathematical theory of communication. Urbana, IL: University of Illinois Press.

[74] Cavalli-Sforza L, Edwards A (1967) Phylogenetic analysis. Models and estimation procedures. Am J Hum Genet 19: 233–257. 6026583 PMC1706274

[75] Nei M (1987) Molecular evolutionary genetics. Columbia University Press, New York.

[76] Sokal RR, Michener CD (1958) A statistical method for evaluating systematic relationships: Univ Kansas Sci. Bull. 38: 1409–1438.

[77] Saitou N, Nei M (1987) The neighbor-joining method: a new method for reconstructing phylogenetic trees. Mol Biol Evol 4: 406–25. 3447015 10.1093/oxfordjournals.molbev.a040454

[78] Liu K, Muse SV (2005) PowerMarker: an integrated analysis environment for genetic marker analysis. Bioinformatics 21: 2128–2129. 15705655 10.1093/bioinformatics/bti282

[79] Hammer Ø, Harper DAT, Ryan PD (2001) *Past*: Paleontological statistics software package for education and data analysis. Palaeontol Electron 4: 9pp.

[80] Yu J, Pressoir G, Briggs WH, Vroh Bi I, Yamasaki M, et al (2006) A unified mixed-model method for association mapping that accounts for multiple levels of relatedness. Nat Genet 38: 203–208. 16380716 10.1038/ng1702

[81] Price AL, Patterson NJ, Plenge RM, Weinblatt ME, Shadick NA, et al (2006) Principal components analysis corrects for stratification in genome-wide association studies. Nature Genetics 38: 904–909. 16862161 10.1038/ng1847

[82] Mezmouk S, Dubreuil P, Bosio M, Décousset L, Charcosset A, et al (2011) Effect of population structure corrections on the results of association mapping tests in complex maize diversity panels. Theor Appl Genet 122: 1149–1160. 21221527 10.1007/s00122-010-1519-y PMC3057001

[83] Pritchard JK, Stephens M, Donnelly P (2000) Inference of population structure from multilocus genotype data. Genetics 155: 945–959. 10835412 10.1093/genetics/155.2.945 PMC1461096

[84] Evanno G, Regnaut S, Goudet J (2005) Detecting the number of clusters of individuals using the software Structure, a simulation study. Mol Ecol 14: 2611–2620. 15969739 10.1111/j.1365-294X.2005.02553.x

[85] Jakobsson M, Rosenberg NA (2007) *Clumpp:* a cluster matching and permutation program for dealing with label switching and multimodality in analysis of population structure. Bioinformatics 23: 1801–1806. 17485429 10.1093/bioinformatics/btm233

[86] Hardy OJ, Vekemans X (2002) *Spagedi*: a versatile computer program to analyze spatial genetic structure at the individual or population levels. Mol Ecol Notes 2: 618–620.

[87] Loiselle BA, Sork VL, Nason J, Graham C (1995) Spatial genetic structure of a tropical understory shrub, *Psychotria officinalis* (Rubiaceae). Am J Bot. 82: 1420–1425.

[88] Bradbury PJ, Zhang ZW, Kroon DE, Casstevens TM, Ramdoss Y, et al (2007) *Tassel*: software for association mapping of complex traits in diverse samples. Bioinformatics 23: 2633–2635. 17586829 10.1093/bioinformatics/btm308

[89] Khadari B, Zine El Aabidine A, Grout C, Ben Sadok I, Doligez A, et al (2010) A Genetic Linkage Map of Olive Based on Amplified Fragment Length Polymorphism, Intersimple Sequence Repeat and Simple Sequence Repeat Markers. J Am Soc Hortic Sci 135: 548–555.

[90] Zine El Aabidine A, Charafi J, Grout C, Doligez A, Santoni S, et al (2010) Construction of a genetic linkage map for the olive based on AFLP and SSR markers. Crop Sci 50: 2291–2302.

[91] Ehrenreich IM, Stafford PA, Purugganan MD (2007) The Genetic architecture of shoot branching in *Arabidopsis thaliana*: A comparative assessment of candidate gene associations *vs*. quantitative trait locus mapping. Genetics 176: 1223–1236. 17435248 10.1534/genetics.107.071928 PMC1894586

[92] Skøt L, Humphreys J, Humphreys MO, Thorogood D, Gallagher J, et al (2007) Association of candidate genes with flowering time and water-soluble carbohydrate content in *Lolium perenne* (L.). Genetics 177: 535–547. 17660575 10.1534/genetics.107.071522 PMC2013705

[93] Grenier C, Bramel-Cox PJ, Noirot M, Prasada Rao KE, Hamon P (2000) Assessment of genetic diversity in three subsets constituted from the ICRISAT sorghum collection using random vs non-random sampling procedures A. Using morpho-agronomical and passport data. Theor Appl Genet 101: 190–196.

[94] Miranda C, Urrestarazu J, Santesteban LG, Royo JB, Uribina V (2010) Genetic diversity and structure in a collection of ancient Spanish pear cultivars assessed by microsatellite markers. J Am Soc Hortic Sci 135: 428–437.

[95] Laucou V, Lacombe T, Dechesne F, Siret R, Bruno JP, et al (2011) High throughput analysis of grape genetic diversity as a tool for germplasm collection management. Theor Appl Genet. 122: 1233–1245. 10.1007/s00122-010-1527-y 21234742

[96] Besnard G, Baradat P, Breton C, Khadari B, Bervillé A (2001) Olive domestication from structure of oleasters and cultivars using nuclear RAPDs and mitochondrial RFLPs. Genet Sel Evol 33: S251–S268.

[97] Pino Del Caprio D, Basnet RK, De Vos RCH, Maliepaard C, Paulo MJ, et al (2011) Comparative methods for association studies: A case study on metabolite variation in *Brassica rapa* core collection. PloS ONE 6: e19624. 21602927 10.1371/journal.pone.0019624 PMC3094343

[98] Jestin C, Lodé M, Vallée P, Domin C, Falentin C, et al (2011) Association mapping of quantitative resistance for *Leptosphaeria maculans* in oilseed rape (*Brassica napus* L.). Mol Breed 27: 271–287.

