# Supplementary figures and images for: Construction of Core Collections Suitable for Association Mapping to Optimize Use of Mediterranean Olive (Olea europaea L.) Genetic Resources
Source: PLoS One. 2013 May 7;8(5):e61265. doi: 10.1371/journal.pone.0061265 (PMC3646834; doi:10.1371/journal.pone.0061265)

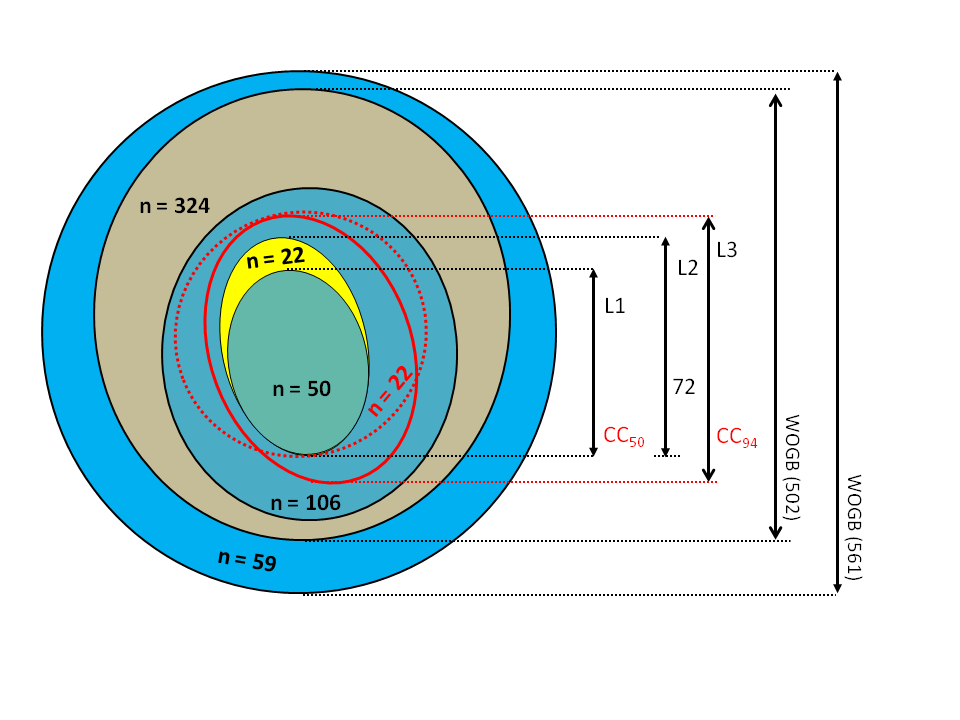

Supplement: Figure S2 — Three different levels proposed for core collections. Level 1 (L1) represents the primary core collection (CC50), which includes the 40 entries selected using the “Sh strategy” implemented in Core Hunter program at 8%, two varieties carrying the two missing cpDNA haplotypes, and 8 non-selected reference varieties among the 14. Level 2 includes accessions carrying alleles observed once (22 genotypes). Level 3 represents final core collections (CC94) constructed by adding a complement of 22 genotypes to the previous 72 among a panel of 106 genotypes to capture the total allelic and phenotypic diversity. (TIF) [file pone.0061265.s002.tif]

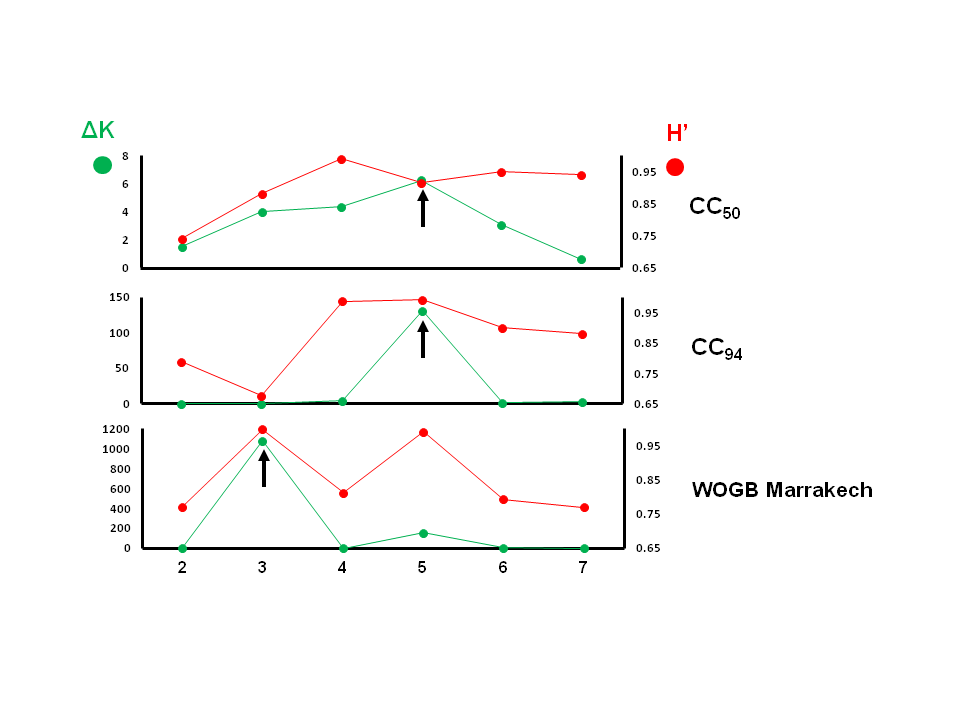

Supplement: Figure S3 — Plot of ad-hoc ΔK measurements and coefficients of similarity ( H′ ) for K between 2 and 7. Arrows indicate the best genetic structure model for both core collections and OWGB Marrakech. According to both parameters, i.e. ΔK and H′, the best genetic structure model was not stable, while it is defined at K = 3 in Marrakech OWGB, indicating the absence of an obvious genetic structure in the core collections (see Figure S3). (TIF) [file pone.0061265.s003.tif]

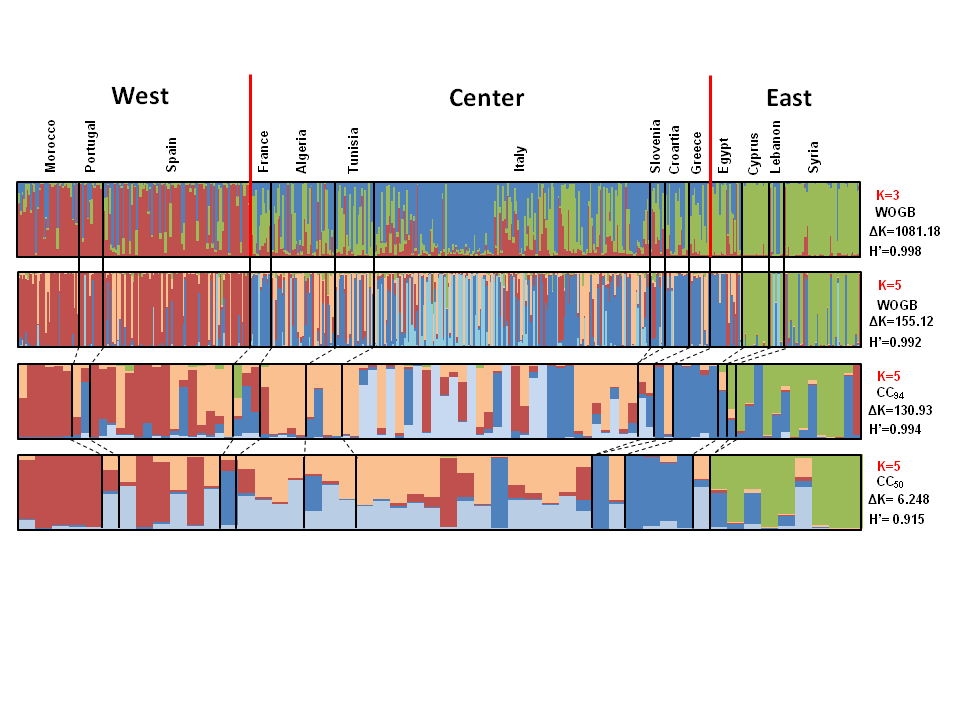

Supplement: Figure S4 — Inferred structure for K = 5 clusters within OWGB Marrakech, CC50, and CC94 core collections. H′ represents the similarity coefficient between runs, and ΔK represents the ad-hoc measure of Evanno et al. [84]. No consistency was observed in genetic structures based on more than three clusters. (TIF) [file pone.0061265.s004.tif]
