# Supplementary material for: Construction of Core Collections Suitable for Association Mapping to Optimize Use of Mediterranean Olive (Olea europaea L.) Genetic Resources
Source: PLoS One. 2013 May 7;8(5):e61265. doi: 10.1371/journal.pone.0061265 (PMC3646834; doi:10.1371/journal.pone.0061265)
Supplement: Table S2 — List of traits, number of trait classes according to standards described by the International Olive Oil Council, and number of varieties with available phenotypic data. The number of varieties differed according to traits indicates that there was missing data, and that not all varieties were completely characterized with the 72 phenotypic traits. (DOC) [file pone.0061265.s006.doc]

**Table** **S2.** List of traits, number of trait classes according to standards described by the International Olive Oil Council, and number of varieties with available phenotypic data. The number of varieties differed according to traits indicates that there was missing data, and that not all varieties were completely characterized with the 72 phenotypic traits.

|  | **Traits** | **Number of trait classes** | **Number of varieties** |
| --- | --- | --- | --- |
| Tree | 1. Vigour | 3 | 347 |
|  | 2. Habit | 3 | 325 |
|  | 3. Canopy density | 3 | 325 |
|  | 4. Inter-node length | 3 | 233 |
| Inflorescence | 1. Inflorescence structure | 2 | 59 |
|  | 2. Inflorescence length | 3 | 342 |
|  | 3. Number of flowers per inflo. | 3 | 344 |
| Leaf | 1. Blade length (mm) | 3 | 342 |
|  | 2. Blade width (mm) | 3 | 340 |
|  | 3. Leaf shape | 3 | 386 |
|  | 4. Longitudinal curvature of blade | 4 | 369 |
| Fruit | 1. Fruit weight (g) | 4 | 377 |
|  | 2. Fruit shape | 3 | 402 |
|  | 3. Symmetry in position A | 3 | 387 |
|  | 4. Situation of maximum transverse diameter in position B | 3 | 385 |
|  | 5. Apex in position A | 2 | 385 |
|  | 6. Base in position A | 2 | 384 |
|  | 7. Nipple | 2 | 379 |
|  | 8. Presence of lenticels | 2 | 382 |
|  | 9. Size of lenticels | 2 | 370 |
|  | 10. Colour at full maturity | 2 | 325 |
|  | 11. Ripeness start | 3 | 352 |
| Endocarp | 1. Stone weight (g) | 3 | 358 |
|  | 2. Stone shape | 4 | 401 |
|  | 3. Symmetry in position A | 3 | 385 |
|  | 4. Symmetry in position B | 2 | 354 |
|  | 5. Situation of maximum transverse diameter in position B | 3 | 384 |
|  | 6. Apex in position A | 2 | 386 |
|  | 7. Base in position A | 3 | 385 |
|  | 8. Surface in position B | 3 | 386 |
|  | 9. Number of grooves | 3 | 368 |
|  | 10. Distribution of grooves | 2 | 360 |
|  | 11. Depth of grooves | 3 | 49 |
|  | 12. Termination of the apex in position A | 2 | 370 |
| Agronomic | 1. Fruit-flesh/pit ratio [fresh weight basis] | 3 | 250 |
|  | 2. Fruit-beginning of first fruiting | 3 | 175 |
|  | 3. Yield | 3 | 293 |
|  | 4. Tree productivity | 4 | 314 |
|  | 5. Purpose | 3 | 406 |
|  | 6. Flower fertility | 4 | 220 |
|  | 7. Oil content [flesh dry weight basis (%)] | 3 | 351 |
|  | 8. Fruit-flesh clinginess to stone | 2 | 78 |
|  | 9. Fruit-flesh consistency at ripening | 3 | 45 |
|  | 10. Rooting ability | 4 | 244 |
|  | 11. Tree-time of flowering | 3 | 173 |
|  | 12. Tree-harvest time | 3 | 239 |
|  | 13. Fruit retention force at ripening | 3 | 176 |
| Oil chemical composition | 1. Oleic acid content | 4 | 149 |
|  | 2. Linoleic acid content | 4 | 148 |
|  | 3. Palmitic acid content | 4 | 145 |
|  | 4. Stearic acid content | 3 | 126 |
|  | 5. Polyphenol content [total] | 3 | 96 |
| Abiotics stress | 1. Cold | 3 | 226 |
|  | 2. Drought | 3 | 184 |
|  | 3. Salinity | 3 | 48 |
|  | 4. Calcareous | 3 | 24 |
|  | 5. Wind | 3 | 45 |
|  | 6. Soil humidity | 3 | 31 |
|  | 7. Air humidity | 3 | 19 |
| Biotics stress | *1. Spilocaea oleagina* | 3 | 201 |
|  | *2. Pseudomonas syringae* | 3 | 174 |
|  | *3. Verticillium dahlia* | 3 | 86 |
|  | 4. Sooty moulds | 3 | 56 |
|  | *5. Cercospora cladosporioides* | 3 | 31 |
|  | *6. Gloesporium olivarum* | 3 | 38 |
|  | *7. Sphaeropsis dalmatica* | 3 | 25 |
|  | *8. Bactrocera oleae* | 3 | 170 |
|  | *9. Prays oleae* | 3 | 60 |
|  | *10. Saissetia oleae* | 3 | 51 |
|  | 11. Strawberry latent ringspot nepovirus | 3 | 27 |
|  | 12. Cucumber mosaic cucumovirus | 3 | 19 |
|  | 13. Cherry leaf roll virus | 3 | 18 |

Data was compiled from olive databases and different national catalogues [68-72].
