# Supplementary material for: Construction of Core Collections Suitable for Association Mapping to Optimize Use of Mediterranean Olive (Olea europaea L.) Genetic Resources
Source: PLoS One. 2013 May 7;8(5):e61265. doi: 10.1371/journal.pone.0061265 (PMC3646834; doi:10.1371/journal.pone.0061265)
Supplement: Table S3 — Genetic parameters of core subsets sampled using four different strategies with the ASLS method at four sample sizes, i.e. 4, 8, 24, and 32%. The CC2-40 core subset (in bold) was chosen as the optimal to construct final core collections. (DOC) [file pone.0061265.s007.doc]

**Table S3.** Genetic parameters of core subsets sampled using four different strategies with the advanced stochastic local search method (ASLS) at four sample sizes, i.e. 4% to 32%. The CC2-40 core subset (in bold) was chosen as the optimal to construct the final core collection.

| ***Sampling Size*** | ***Subset code*** | ***Method/allocation strategy*** | ***Cv (%)*** | ***D_CE_ (****±****SD)*** | | ***He*** | ***Sh*** | **# Trait classes (%)** | | **# Haplotype (%)** | |
| --- | --- | --- | --- | --- | --- | --- | --- | --- | --- | --- | --- |
|  |  | OWGB | 279 (100) | 0.745 (±0.092) | | 0.728 | 4.524 | 213 | | 12 | |
| 4 % (20 entries) | CC1-20 | ASLS/D_CE_^1^ | 171 (61.3)* | 0.865 (±0.05) | | 0.817* | 4,807 | 185 (86.8) | | 6 (50) | |
|  | CC2-20 | ASLS/Sh^1^ | 193 (69.1)* | 0.853 (±0.054) | | 0.817* | 4.877 | 162 (76) | | 9 (75) | |
|  | CC3-20 | ASLS/He^1^ | 175 (62.7)* | 0.859 (±0.053) | | 0.827* | 4,838 | 169 (79.3) | | 7 (58.3) | |
|  | CC4-20 | ASLS/multi^2^ | 199 (71.3)* | 0.851 (±0.05) | | 0.813* | 4.871 | 174 (81.7) | | 10 (83.3) | |
| 8% (40 entries) |  |  |  |  | |  |  |  | |  | |
|  | CC1-40 | ASLS/D_CE_^1^ | 210 (75.2)* | 0.849 (±0.06) | | 0.816* | 4,861 | 196 (92) | | 9 (75) | |
|  | **CC2-40** | **ASLS/Sh^1^** | **225 (80.6)** | **0.838 (±0.063)** | | **0.814*** | **4.889** | **195 (91.5)** | | **10 (83.3)** | |
|  | CC3-40 | ASLS/He^1^ | 201 (72)* | 0.842 (±0.076) | | 0.824* | 4,853 | 188 (88) | | 10 (83.3) | |
|  | CC4-40 | ASLS/multi^2^ | 231 (82.8) | 0.832 (±0.06) | 0.806* | | 4.881 | | 191 (89.6) | | 10 (83.3) |
| 24% (120 entries) |  |  |  |  | |  |  |  | |  | |
|  | CC1-120 | ASLS/D_CE_^1^ | 249 (89.2) | 0.821 (±0.068) | | 0.797 | 4,794 | 206 (96.7) | | 12 (100) | |
|  | CC2-120 | ASLS/Sh^1^ | 262 (93.9) | 0.812 (±0.067) | | 0.798 | 4.823* | 206 (97.6) | | 11 (91.6) | |
|  | CC3-120 | ASLS/He^1^ | 245 (87.8) | 0.816 (±0.07) | | 0.804* | 4,799* | 203 (95.3) | | 12 (100) | |
|  | CC4-120 | ASLS/multi^2^ | 279 (100) | 0.809 (±0.069) | | 0.788 | 4.796 | 209 (98.1) | | 11 (91.6) | |
| 32% (160 entries) |  |  |  |  | |  |  |  | |  | |
|  | CC1-160 | ASLS/D_CE_^1^ | 264 (94.6) | 0.811 (±0.068) | | 0.788 | 4,763* | 208 (97.6) | | 12 (100) | |
|  | CC2-160 | ASLS/Sh^1^ | 269 (96.4) | 0.804 (±0.07) | | 0.789 | 4.786* | 210 (98.5) | | 12 (100) | |
|  | CC3-160 | ASLS/He^1^ | 256 (91.7) | 0.806 (±0.072) | | 0.794 | 4,762* | 210 (98.6) | | 12 (100) | |
|  | CC4-160 | ASLS/multi^2^ | 279 (100) | 0.799 (±0.073) | | 0.78 | 4.755 | 209 (98.1) | | 12 (100) | |

*Cv:* allelic coverage or number of alleles, *D_CE_:* average genetic distance of Cavalli-sforza and Edwards, *SD:* standard deviation, *Sh:* Shannon-Weaver diversity index, *He:* Nei diversity index, *Na:* number of alleles,. ^1^Each selection parameter was optimized independently by performing 20 runs with 100% weight given to each parameter, respectively. ^2^Twenty independent runs were performed with equal weight given to each of the four parameters simultaneously. *Statistically significant difference (*p<0.05*) using the Mann-Whitney test to assess differences between each core subset and OWGB Marrakech.
