# Supplementary material for: Construction of Core Collections Suitable for Association Mapping to Optimize Use of Mediterranean Olive (Olea europaea L.) Genetic Resources
Source: PLoS One. 2013 May 7;8(5):e61265. doi: 10.1371/journal.pone.0061265 (PMC3646834; doi:10.1371/journal.pone.0061265)
Supplement: Text S1 — Protocols of nuclear and chloroplast loci analyses. (DOC) [file pone.0061265.s009.doc]

**Text S1**. Protocols for nuclear and chloroplast loci analyses

1. **Nuclear SSR loci**

Seventy nuclear SSR loci were selected according to their level of polymorphism and reproducibility, as well as by their easy and accurate scoring based on previous studies: DCA01, DCA03, DCA04, DCA05, DCA08, DCA09, DCA11, DCA15, DCA18 [1], GAPU59, GAPU71A, GAPU71B [2], UDO36 [3], EMO03, EMO90 [4], and PA(TT)2 [5]. PCR amplification was carried out in a total volume of 20 µl containing 20 ng of genomic DNA, 1x PCR buffer, 1.5 mM MgCl2, 0.2 M of each dNTP, 0.1 units of Taq DNA polymerase, and 2 pmol of forward (5’-end labeled; Table S5) and unlabeled reverse primers. Amplifications were performed in a thermal cycler (Mastercycler ep gradient S) with 94°C at 5 min, followed by 35 denaturation cycles at 94°C for 30 s, T°C annealing for 1 min (Table S5) and 72°C extension for 1 min. A 10 min extension at 72°C was used post-thermocycling. Amplified products corresponding to each combination, as listed in Table S5, were diluted at 1:9 with dH_2_O and 0.5 µl of the solution was then prepared in 17.5 µl of dH_2_O and 0.5 µl of GeneScan 400 HD-Rox as internal standard, which was then run on an automatic capillary sequencer (ABI 3130 Genetic Analyzer Applied Biosystems, Foster City, CA, USA). Chromatograms were then analysed with GeneMapper 3.7 software (Applied Biosystems).

**Table S5.** List of combinations of nuclear SSR loci used for characterizing OWGB Marrakech.

| **Combinaison** | **SSR Loci** | **Fluorescent dye** | ****T°C*** |
| --- | --- | --- | --- |
| 1 | DCA04*^a^* | HEX | *57* |
|  | DCA09*^a^* | NED |  |
|  | DCA15*^a^* | FAM |  |
| 2 | DCA03*^a^* | NED | *50* |
|  | DCA05*^a^* | FAM |  |
|  | UDO36*^b^* | HEX | *57* |
| 3 | DCA08*^a^* | FAM | 55 |
|  | DCA18*^a^* | NED |  |
|  | GAPU59*^c^* | HEX |  |
| 4 | EMO90*^d^* | FAM | 57 |
|  | GAPU71B*^c^* | FAM |  |
|  | DCA11*^a^* | HEX |  |
| 5 | DCA01*^a^* | FAM | 57 |
|  | DCA14*^a^* | NED |  |
|  | EMO03*^d^* | HEX |  |
| 6 | GAPU71A*^c^* | FAM | 57 |
|  | PA(ATT)2 ^e^ | HEX |  |

*^*^* Annealing temperature.

*^a^*Sefc et al., 2000.

*^b^*Cipriani et al., 2002.

*^c^*Carriero et al., 2002.

*^d^*De la Rosa et al., 2002.

*^e^*Saumitou-Laprade et al., 2000.

**2- Chloroplast loci**

Plastid DNA (or *cpDNA*) was characterized using 37 polymorphic loci and two cleaved amplified polymorphism sites (CAPS-*XapI* and CAPS-*EcoRI)* [6]. Six multiplexes of six loci each were amplified in a 25 µl total reaction volume using an 18-bp tail of M13 on the forward primer according to the protocol described by Besnard et al. [6], and one locus (locus 19) was amplified separately without the M13 primer. The PCR products of the six multiplexes were mixed together (with no overlap for allele size between loci in a given colour), and locus 19 was added to the two CAPS for a second combination of three loci. Both combinations were separated using an automatic capillary sequencer (ABI prism 3130XL Genetic Analyzer Applied Biosystems, Foster City, CA, USA) and chromatograms were then analysed with GeneMapper 3.7 software (Applied Biosystems).

**References**

1. Sefc KM, Lopes MS, Mendonc¸a D, Rodrigues Dos Santos M, Da Ca´mara Machado L (2000) Identification of microsatellites loci in Olive (Olea europaea L.) and their characterization in Italian and Iberian trees. Mol Ecol 9:171–1193.

2. Carriero F, Fontanazza G, Cellini F, Giorio G (2002) Identification of simple sequence repeats (SSRs) in olive (Olea europaea L.). Theor Appl Genet 104:301–307.

3. Cipriani G, Marrazzo MT, Marconi R, Cimato A, Testolin R (2002) Microsatellite markers isolated in olive (Olea europaea L.) are suitable for individual fingerprinting and reveal polymorphism within ancient cultivars. Theor Appl Genet 104:223–228.

4. De La Rosa R, James CM, Tobutt KR (2002) Isolation and characterization of polymorphic microsatellites in olive (Oleaeuropaea L.) and their transferability to other genera in the Oleaceae. Mol Ecol Notes 2:265–267.

5. Saumitou-Laprade P, Vassiliadis C, Epplen JT, Hardt C (2000) Isolation of microsatellite loci for paternity testing in *Phillyrea angustifolia* L. (Oleaceae). Mol Ecol 9:112-114.

6. Besnard G, Hernandez P, Khadari B, Dorado G, Savolainen V (2011) Genomic profiling of plastid DNA variation in the Mediterranean olive tree. BMC Plant Biology 11:80.
