# Supplementary material for: Construction of Core Collections Suitable for Association Mapping to Optimize Use of Mediterranean Olive (Olea europaea L.) Genetic Resources
Source: PLoS One. 2013 May 7;8(5):e61265. doi: 10.1371/journal.pone.0061265 (PMC3646834; doi:10.1371/journal.pone.0061265)
Supplement: Text S2 — Genetic analysis of OWGB Marrakech. (DOC) [file pone.0061265.s010.doc]

**Text S2.** Genetic analysis of WOGB Marrakech

**1. Nuclear SSR characterization**

**1.1. Data analysis**

The number of alleles per locus (*Na*), allelic frequencies (*p_i_*), polymorphism information content (PIC) [1], expected (*He*) [2] and observed heterozygosity (*Ho*) were estimated using the Excel Microsatellite Toolkit v3.1 [3]. The discrimination power of each SSR locus (*D_j_*) was computed as defined by Tessier et al. [4].

- 1. **Nuclear SSR polymorphism**

Using 17 nuclear SSR loci, the 561 accessions were classified into 502 distinct SSR profiles. A total of 279 alleles were revealed with frequencies ranging from 70% to 0.1% (observed once). The number of alleles ranged from 5 to 32 per locus with an average of 16.4 alleles per locus (Table S6). The allele size ranged from 103 bp at the PA(TTA)2 locus to 275 bp at the DCA01 locus. Among the 279 alleles detected, 152 alleles (54.5%) had a frequency of less than 1% (observed 10 times) including 42 alleles (15%) observed once in 36 genotypes. Two among the 14 reference cultivars considered in the present work were each found to be carrying unique alleles; “Amphisis” and “Chemlal de Kabylie” cultivars.

**Table S6.** Genetic parameters of the 17 SSR loci used to characterize WOGB Marrakech. Number of alleles (*Na*)*,* expected (*He*), observed heterozygosity (*Ho*), polymorphism information content (*PIC*), and power of discrimination (*Dj*)

| **Loci** | ***Na*** | **Size** | **<0.1^1^** | ***He*** | ***Ho*** | ***PIC*** | ***Dj*** |
| --- | --- | --- | --- | --- | --- | --- | --- |
| DCA01*^a^* | 20 | 173-275 | 8 | 0.601 | 0.708 | 0.55 | 0.841 |
| DCA03*^a^* | 14 | 229-265 | 1 | 0.871 | 0.89 | 0.857 | 0.994 |
| DCA04*^a^* | 32 | 117-194 | 5 | 0.858 | 0.621 | 0.844 | 0.994 |
| DCA05*^a^* | 14 | 192-218 | 2 | 0.483 | 0.49 | 0.469 | 0.736 |
| DCA08*^a^* | 21 | 125-168 | 5 | 0.824 | 0.921 | 0.802 | 0.977 |
| DCA09*^a^* | 24 | 162-219 | 2 | 0.876 | 0.928 | 0.864 | 0.988 |
| DCA11*^a^* | 25 | 126-196 | 3 | 0.82 | 0.726 | 0.797 | 0.986 |
| DCA14*^a^* | 16 | 165-195 | 1 | 0.704 | 0.78 | 0.677 | 0.948 |
| DCA15*^a^* | 8 | 231-266 | 3 | 0.627 | 0.652 | 0.563 | 0.798 |
| DCA18*^a^* | 19 | 155-207 | 1 | 0.831 | 0.836 | 0.812 | 0.965 |
| UDO36*^b^* | 16 | 134-168 | 1 | 0.735 | 0.693 | 0.691 | 0.961 |
| GAPU59*^c^* | 12 | 207-241 | 2 | 0.636 | 0.697 | 0.592 | 0.875 |
| GAPU71A*^c^* | 15 | 124-255 | 6 | 0.454 | 0.555 | 0.403 | 0.642 |
| GAPU71B*^c^* | 13 | 117-166 | -- | 0.828 | 0.903 | 0.806 | 0.987 |
| EMO03*^d^* | 12 | 204-218 | -- | 0.781 | 0.769 | 0.751 | 0.913 |
| EMO90*^d^* | 13 | 163-210 | 2 | 0.683 | 0.775 | 0.649 | 0.935 |
| PA(ATT)2 ^e^ | 5 | 103-121 | -- | 0.732 | 0.873 | 0.691 | 0.889 |
| **Total** | **279** | **----** | **42** | **----** | **----** | **----** | **----** |
| **Mean** | **16.41** | **----** | **2.47** | **0.726** | **0.754** | **0.695** | **0.907** |

*^a^*Sefc et al., 2000 [5]

*^b^*Cipriani et al., 2002 [6]

*^c^*Carriero et al., 2002 [7]

*^d^*De la Rosa et al., 2002 [8]

*^e^*Saumitou-Laprade et al., 2000 [9]

^1^number of allele observed once.

Among the 125,751 pairwise comparisons obtained from the 502 SSR profiles, 79 pairwise profiles were closely related and differed by less than 3 dissimilar alleles, whereas the remaining were distinguished by 4 to 57 dissimilar alleles (91%; Figure S5), thus 457 genotypes were detected by pooling closely related genotypes under a common single genotype.


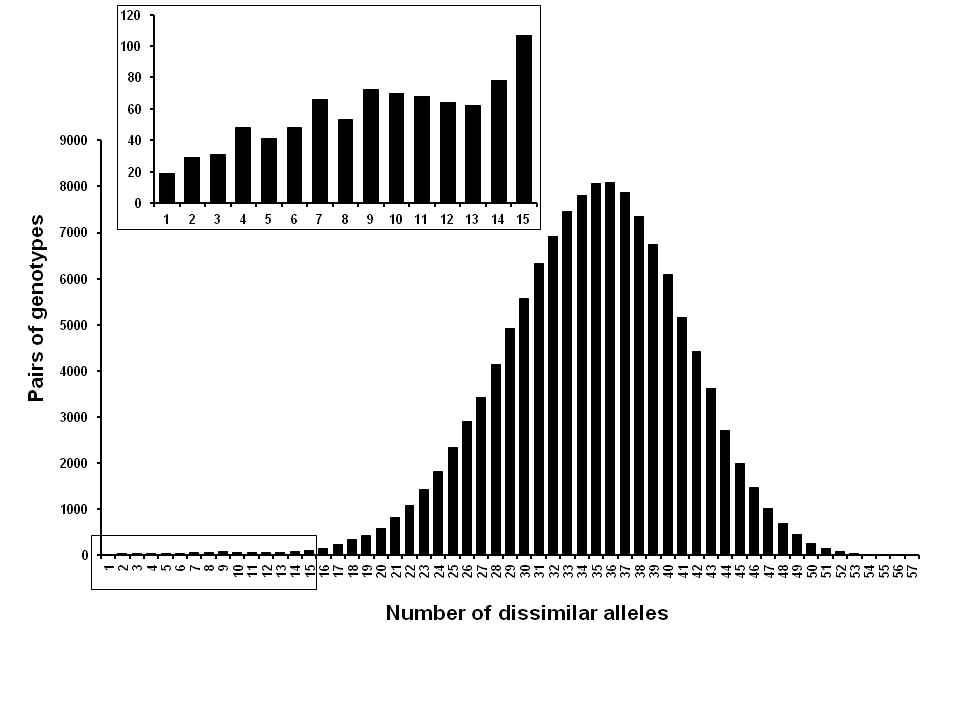


**Figure S5.** Frequency distribution of allele dissimilarity for all pairwise combinations from the 502 SSR profiles revealed in WOGB Marrakech.

For the 502 SSR profiles, the observed heterozygosity (*Ho*) ranged from 0.49 to 0.928 and the expected heterozygosity (*He*) ranged from 0.454 to 0.876, with an average of 0.754 and 0.726, respectively. Eight out of the 17 microsatellite loci used had PIC values above 0.7, while the GAPU71A locus had the lowest value (0.403). For each SSR locus, the discrimination power (*Dj*) ranged from 0.994 at DCA03 and DCA04 to 0.642 at GAPU71A, with a mean of 0.907 (Table S6). The number of single genotypes identified per locus ranged from 16 (DCA15 and PA(TTA)2) to 95 (DCA04), with an average of 43.8 genotypes per locus.

- 1. **Genetic structure of WOGB Marrakech**

Model-based Bayesian clustering implemented in the *Structure* program [10] revealed the presence of three gene pools (eastern, western and central Mediterranean; Figure S6). No significant differences in allelic richness were observed between the three gene pools (pairwise Mann-Whitney test, using 17 SSR loci computed at 91 individuals of standardized G value using the rarefaction method, ADZE program) [11]. A low Nei diversity index was noted in the western gene pool, while the average genetic distance (*D_CE_*) was similar within each gene pool (Table S7). Higher genetic differentiation (*Fst*) was revealed between eastern and western cultivars than between central/western and central/eastern cultivars (Table S8).

**Table S7**. Number of genotypes and genetic parameters for each of the three gene pools: number of alleles (*Na*), Nei diversity index (*He*), Shannon-Weaver diversity index (*Sh*), Cavalli-Sforza and Edwards genetic distance (*D_CE_*)

| **Gene pool** | **# genotypes** | ***Na*** | ***He*** | ***Sh*** | ***D_CE_*** |
| --- | --- | --- | --- | --- | --- |
| **Eastern** | 91 | 168 | 0.705 | 4.427 | 0.732 (±0.116) |
| **Western** | 271 | 186 | 0.676 | 4.292 | 0.675 (±0.12) |
| **Central** | 140 | 238 | 0.732 | 4.502 | 0.75 (±0.088) |

**Table S8***.* Pairwise *Fst* between gene pools using 100 permutations

|  | **Central** | **Eastern** |
| --- | --- | --- |
| **Eastern** | 0.032 |  |
| **Western** | 0.034 | 0.055 |

1. **Chloroplast loci characterization**

The set of the 37 chloroplast SSRs and the two CAPS markers revealed the presence of 12 haplotypes in the collection (Table S9; Figure S6). A new haplotype (E1.12) was observed once and not reported in Besnard et al. [12]. The most frequent haplotype (E1.1) had a frequency of 83.2%. Except for E1.1 haplotype, only one showed a frequency of over 7% (E1.2) and the remaining were less frequent, while some of them were detected only once (E1.10, E1.12, E2.3, and L1.1). The L1.1 haplotype was revealed in *laperrinei* subspecies [13], and was observed in the “Doukar” Tunisian cultivar, suggesting introgression between *laperrinei* and *europaea* subspecies and a contribution of wild olive relatives in cultivar selection (Besnard et al., in prep). The number of haplotypes per country ranged from one (E1.1 in Cyprus, Croatia and Slovenia) to six haplotypes in Italy (Table S9; Figure S6).

**Table S9.** Number and frequency of each of the 12 haplotypes (maternal lineages) revealed in the 502 genotypes per country and per geographical zone. Numbers in brackets are the frequencies of each haplotype.

|  | **E1.1** | **E1.2** | **E1.3** | **E1.10** | **E1.12** | **L1.1** | **E2.1** | **E2.2** | **E2.3** | **E3.1** | **E3.2** | **E3.3** | **Total** |
| --- | --- | --- | --- | --- | --- | --- | --- | --- | --- | --- | --- | --- | --- |
| Morocco | 34 (6.8) | 1 (0.2) | 1 (0.2) |  |  |  |  |  |  |  |  | 1 (0.2) | 37 (7.4) |
| Portugal | 13 (2.6) | 1 (0.2) |  |  |  |  |  |  |  |  |  |  | 14 (2.8) |
| Spain | 80 (16) |  |  |  |  |  | 1 (0.2) |  | 1 (0.2) | 7 (1.4) |  |  | 89 (17.8) |
| **Western Mediterranean** | **127 (25.4)** | **2 (0.4)** | **1 (0.2)** |  |  |  | **1 (0.2)** |  | **1 (0.2)** | **7 (1.4)** |  | **1 (0.2)** | **140 (28)** |
|  |  |  |  |  |  |  |  |  |  |  |  |  |  |
| Algeria | 24 (4.8) | 3 (0.6) |  |  |  |  | 7 (1.4) |  |  |  | 2 (0.4) | 2 (0.4) | 38 (7.6) |
| Croatia | 14 (2.8) |  |  |  |  |  |  |  |  |  |  |  | 14 (2.8) |
| France | 8 (1.6) |  |  |  |  |  | 2 (0.4) |  |  | 1 (0.2) |  |  | 11 (2.2) |
| Greece | 12 (2.4) |  |  |  |  |  |  |  |  | 1 (0.2) |  |  | 13 (2.6) |
| Italy | 146 (29.2) | 11 (2.2) | 1 (0.2) |  |  |  | 1 (0.2) | 3 (0.6) |  |  | 1 (0.2) |  | 163 (32.6) |
| Slovenia | 9 (1.8) |  |  |  |  |  |  |  |  |  |  |  | 9 (1.8) |
| Tunisia | 16 (3.2) | 6 (1.2) |  |  |  | 1 (0.2) |  |  |  |  |  |  | 23 (4.6) |
| **Central Mediterranean** | **229 (45.8)** | **20 (4)** | **1 (0.2)** |  |  | **1 (0.2)** | **10 (2.0)** | **3 (0.6)** |  | **2 (0.4)** | **3 (0.6)** | **2 (0.4)** | **271 (54.2)** |
|  |  |  |  |  |  |  |  |  |  |  |  |  |  |
| Cyprus | 16 (3.2) |  |  |  |  |  |  |  |  |  |  |  | 16 (3.2) |
| Egypt | 12 (2.4) | 6 (1.2) |  |  |  |  | 1 (0.2) |  |  |  |  |  | 19 (3.8) |
| Lebanon | 9 (1.8) |  |  |  |  |  |  |  |  |  |  |  | 9 (1.8) |
| Syria | 23 (4.6) | 10 (2) | 12 (2.4) | 1 (0.2) | 1 (0.2) |  |  |  |  |  |  |  | 47 (9.4) |
| **Eastern Mediterranean** | **60 (12)** | **16 (3.2)** | **12 (2.4)** | **1 (0.2)** | **1 (0.2)** |  | **1 (0.2)** |  |  |  |  |  | **91 (18.2)** |
|  |  |  |  |  |  |  |  |  |  |  |  |  |  |
| **Total** | **416 (83.2)** | **38 (7.6)** | **14 (2.8)** | **1 (0.2)** | **1 (0.2)** | **1 (0.2)** | **12 (2.4)** | **3 (0.6)** | **1 (0.2)** | **9 (1.8)** | **3 (0.6)** | **3 (0.6)** | **502 (100)** |


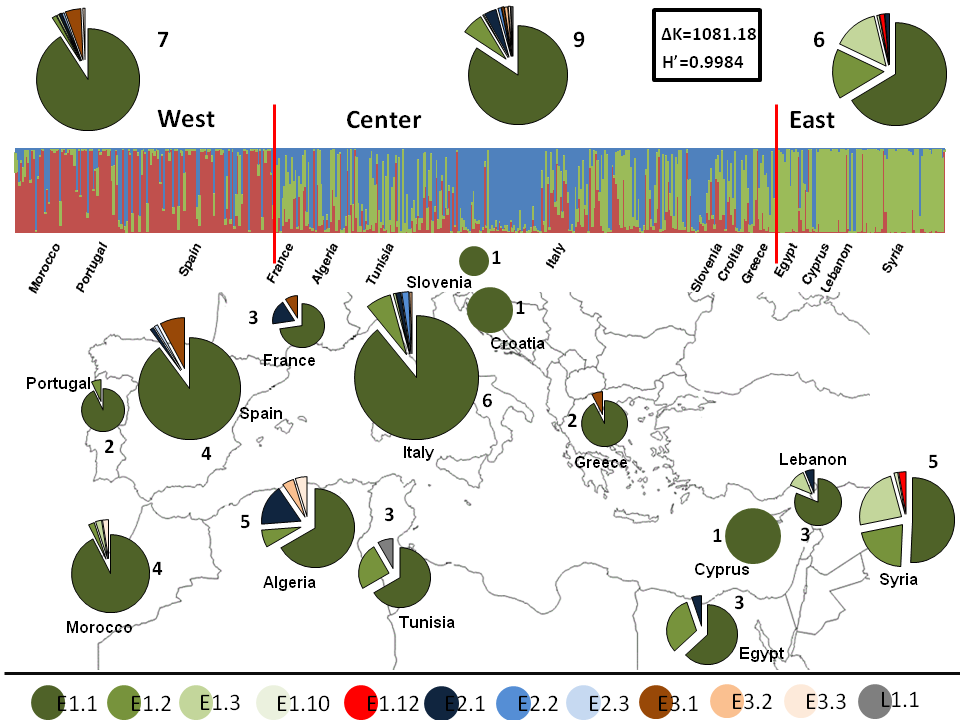


**Figure S6.** Inferred structure within the 502 genotypes from WOGB Marrakech at K=3, and distribution of *cpDNA* haplotypes among 14 countries and among the 3 gene pools. Numbers indicate the number of haplotypes observed for each country and for each gene pool. The set of *cpDNA* markers revealed 12 haplotypes in WOGB Marrakech with one highly frequent one (E1.1, 83.2%).

**References**

1. Botstein D, White RL, Skolnick M, Davis RW (1980) Construction of a genetic linkage map in man using restriction of fragment length polymorphism. Amer J Hum Genet 32: 314-331.

2. Nei M (1987) Molecular evolutionary genetics. Columbia University Press, New York.

3. Park SDE (2001) Trypanotolerance in West African cattle and the population genetic effects of selection. PhD Thesis, Univ. of Dublin.

4. Tessier C, David J, This P, Boursiquot JM, Charrier A (1999) Optimization of the choice of molecular markers for varietal identification in *Vitis vinifera* L. Theor Appl Genet 98: 171-177.

5. Sefc KM, Lopes MS, Mendonc¸a D, Rodrigues Dos Santos M, Da Ca´mara Machado L (2000) Identification of microsatellites loci in Olive (Olea europaea L.) and their characterization in Italian and Iberian trees. Mol Ecol 9:171–1193.

6. Cipriani G, Marrazzo MT, Marconi R, Cimato A, Testolin R (2002) Microsatellite markers isolated in olive (Olea europaea L.) are suitable for individual fingerprinting and reveal polymorphism within ancient cultivars. Theor Appl Genet 104:223–228.

7. Carriero F, Fontanazza G, Cellini F, Giorio G (2002) Identification of simple sequence repeats (SSRs) in olive (Olea europaea L.). Theor Appl Genet 104:301–307.

8. De La Rosa R, James CM, Tobutt KR (2002) Isolation and characterization of polymorphic microsatellites in olive (Oleaeuropaea L.) and their transferability to other genera in the Oleaceae. Mol Ecol Notes 2:265–267.

9. Saumitou-Laprade P, Vassiliadis C, Epplen JT, Hardt C (2000) Isolation of microsatellite loci for paternity testing in *Phillyrea angustifolia* L. (Oleaceae). Mol Ecol 9:112-114.

10. Pritchard JK, Stephens M, Donnelly P (2000) Inference of population structure from multilocus genotype data. Genetics 155: 945-959.

11. Szpiech ZA, Jakobsson M, Rosenberg NA (2008) ADZE: a rarefaction approach for counting alleles private to combinations of populations. Bioinformatics 24: 2498-2504.

12. Besnard G, Hernandez P, Khadari B, Dorado G, Savolainen V (2011) Genomic profiling of plastid DNA variation in the Mediterranean olive tree. BMC Plant Biology 11:80.

13. Besnard G, Christin PA, Baali-Cherif D, Bouguedoura N, Anthelme F (2007) Spatial genetic structure in the Laperrine's olive (Olea europaea subsp. laperrinei), a long-living tree from the central Saharan mountains. Heredity 99:649–657.
